# A Sensitive Ratiometric Thermometer Constructed by AIEgen‐Based Mixed Lanthanide MOF for Intracellular Temperature Mapping

**DOI:** 10.1002/advs.202510147

**Published:** 2025-08-12

**Authors:** Zhijia Li, Jieping Zhang, Zhiyuan Wu, Hang Lei, Yunfang Zhao, Wenqiu Qi, Xue Gao, Feilong Jiang, Yongsheng Liu, Lian Chen, Maochun Hong

**Affiliations:** ^1^ Fujian Science and Technology Innovation Laboratory for Optoelectronic Information of China Fuzhou Fujian 350108 China; ^2^ State Key Laboratory of Structure Chemistry Fujian Institute of Research on the Structure of Matter Chinese Academy of Sciences Fuzhou 350002 China; ^3^ College of Chemistry and Materials Science Fujian Normal University Fuzhou Fujian 350007 China

**Keywords:** bioimaging, intracellular temperature mapping, metal–organic frameworks, ratiometric fluorescence thermometer

## Abstract

Accurate temperature sensing at the subcellular level is of great significance for gaining insights into a wide range of biological processes. Aggregation‐induced emission (AIE) is a promising photophysical phenomenon in which the luminescence intensity is highly related to the degree of restriction of intramolecular motions. By combining AIE luminogen (AIEgen) ligands with mixed lanthanide ions, an AIEgen‐based mixed lanthanide MOF (LnMOF) can be synthesized. In this unique MOF matrix, the vibrations of pendant phenyl rings in the AIEgen ligands are particularly sensitive to temperature changes, rendering the mixed LnMOF highly sensitive as a ratiometric thermometer. Within the physiological temperature range (291–321 K), the maximum relative sensitivity (S_m_) is ≈7.32% K^−1^ at 321 K, demonstrating superior performance compared to reported ratiometric luminescent MOFs. With the aim of detecting intracellular temperature, the mixed LnMOF at the nanoscale is synthesized and further modified, enabling the visual detection of intracellular temperature in L929 cells and HeLa cells. This work demonstrates the first example that MOF‐based ratio thermometers can be practically used in living cells.

## Graphical abstract

Temperature is a crucial fundamental physical parameter involved in all life activities. An AIEgen‐based mixed lanthanide MOF is synthesized. In the physiological temperature range, this MOF shows superior performance. To achieve the detection of intracellular temperature, a nanoscale mixed LnMOF is synthesized and further modified to enable the visual detection of intracellular temperature in L929 cells and HeLa cells.

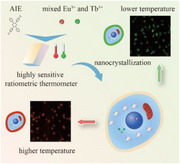

## 1 Introduction

As a fundamental physiological parameter, temperature significantly governs cellular activities by modulating metabolic processes, thereby critically impacting cell viability, functionality, and behavior.^[^ ^1^, ^2^, ^3^, ^4^, ^5^ ^]^ Biological processes are extremely temperature‐sensitive, and even slight deviations in temperature can markedly perturb cellular activities.^[^ ^6^, ^7^, ^8^ ^]^ Enzyme activity is highly sensitive to temperature fluctuation and some gene expression events are regulated by cellular temperature, which in turn affects substance synthesis and energy metabolism.^[^ ^9^, ^10^, ^11^ ^]^ Abnormal temperature changes are often associated with pathological phenomena. For example, inflammation, tumorigenesis, viral infections, and immune responses are typically characterized by elevated temperatures.^[^ ^12^, ^13^, ^14^, ^15^ ^]^ Precise regulation of temperature is also important for disease treatment, including photothermal therapy,^[^ ^16^, ^17^, ^18^ ^]^ magnetic hyperthermia,^[^ ^19^, ^20^ ^]^ and microwave thermotherapy.^[^ ^21^, ^22^ ^]^ Accurate analysis and detection of temperature at the subcellular level is not only essential for understanding diverse physiological processes associated with heat in cells but also provides valuable insights for disease diagnosis and treatment.

Among various types of thermometers, fluorescent thermometers have garnered widespread interest for intracellular temperature monitoring or imaging, owing to their superior spatial resolution, high temperature resolution, and rapid signal feedback.^[^ ^23^, ^24^, ^25^, ^26^, ^27^, ^28^ ^]^ Materials used in the field include upconversion nanoparticles, green fluorescent protein, organic molecules, quantum dots, and nanodiamonds.^[^ ^14^, ^15^, ^29^, ^30^, ^31^ ^]^ The measurement of fluorescence intensity allows for the rapid acquisition of spatial information with high resolution through a relatively straightforward optical setup.^[^ ^32^, ^33^, ^34^, ^35^ ^]^ Nevertheless, their accuracy is often susceptible to external factors such as the concentration of the probes, inhomogeneous particle distribution, and fluctuations in illumination power.^[^ ^36^, ^37^, ^38^ ^]^ Ratiometric intensity‐based thermometers leverage the temperature‐responsive intensity ratio of two emissions to detect temperature, offering a more reliable and accurate outcome.^[^ ^39^, ^40^, ^41^, ^42^ ^]^ Furthermore, the dual‐emission characteristic and their variable intensity ratio often lead to emission color changes of ratiometric thermometers as temperature varies, thereby enabling color‐based intracellular temperature mapping that is readily discernible to the naked eyes.^[^ ^30^, ^43^ ^]^ Lanthanide metal–organic frameworks (LnMOFs) combine the structural versatility of MOFs with the distinctive luminescent properties of lanthanide ions, such as narrow emission bands, high quantum yields, and exceptional resistance to photodegradation.^[^ ^44^, ^45^, ^46^ ^]^ The similar atomic sizes and electronic structures of various lanthanide ions enable the incorporation of different lanthanide ions into one framework, which greatly facilitates the design and construction of ratiometric thermometers.^[^ ^47^, ^48^, ^49^ ^]^ Hitherto, LnMOF‐based ratiometric thermometers have not been employed for intracellular temperature sensing. To this end, two key challenges that remain to be addressed are: i) insufficient sensitivity of the MOF‐based ratiometric thermometers within the physiological temperature range, and ii) the difficulty in downscaling these thermometers into submicron or nanoscale dimensions.

**Figure 1:**
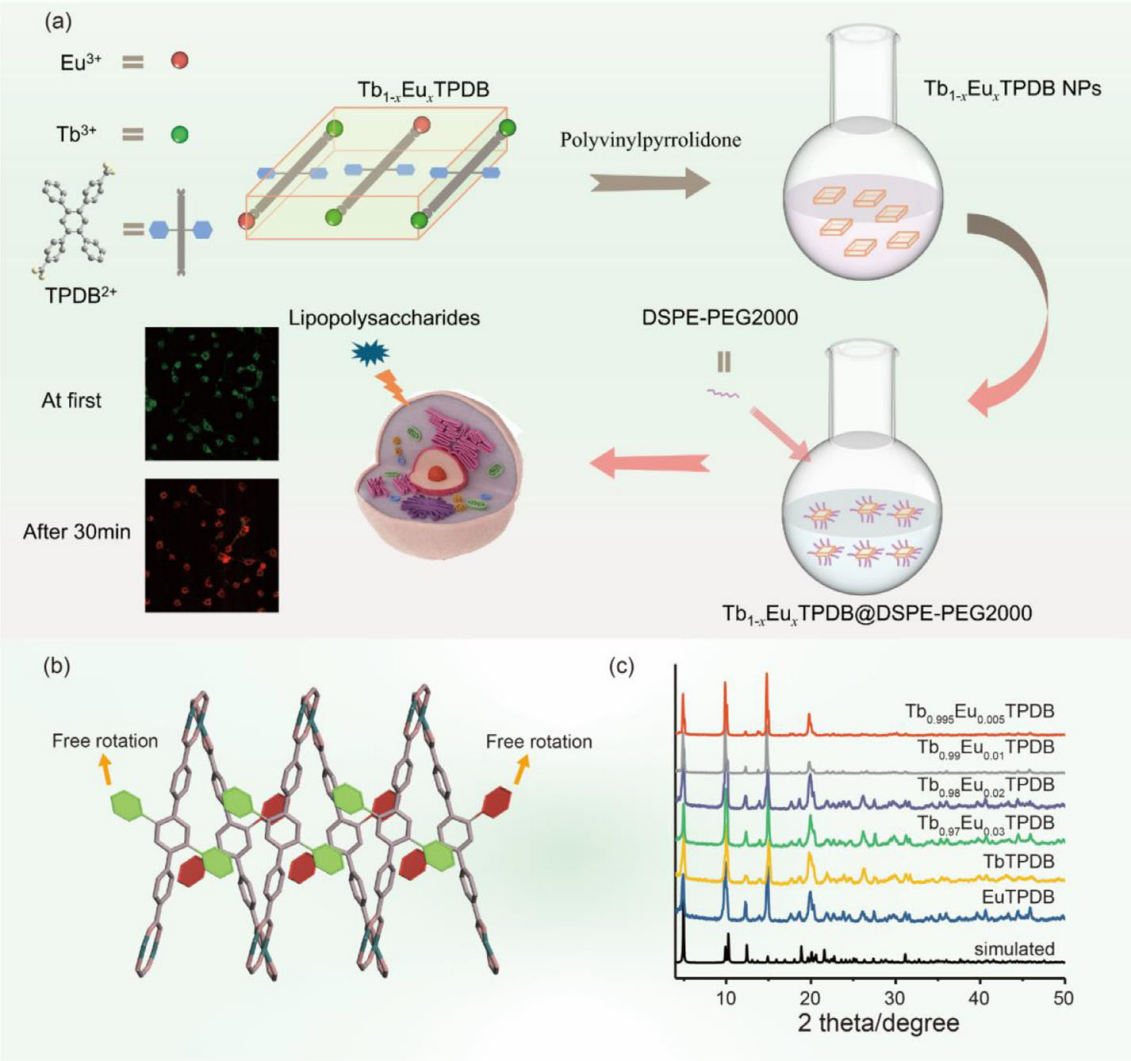
a) Schematic illustration of the synthesis of Tb_1‐_ *_x_* Eu*_x_* TPDB and Tb_0.98_Eu_0.02_TPDB@DSPE‐PEG2000 for intercellular temperature detection. b) The unsubstituted phenyls in the structure of LnTPDB. c) PXRD patterns of LnTPDB with different molar ratios of lanthanides.

Aggregation‐induced emission luminogens (AIEgens) emit non‐ or weak‐ luminescence in the solution state but intense luminescence in the aggregated/solid states due to the restriction of intramolecular motions (RIM).^[^ ^50^, ^51^, ^52^, ^53^, ^54^ ^]^ As temperature exerts a significant influence on molecular motions, the emissions of AIEgens are anticipated to exhibit highly sensitive responses toward temperature changes.^[^ ^55^, ^56^ ^]^ In this study, we focus on improving the sensitivity of ratiometric thermometers for bioimaging applications by incorporating an AIEgen ligand (2′,5′‐diphenyl‐[1,1′:4′,1″‐terphenyl]‐4,4″‐dicarboxylic acid, H_2_TPDB) with pendant phenyl rings into a mixed lanthanide MOF (named Tb_1‐_ *_x_* Eu*_x_* TPDB) (**Figure** **1**a). Tb^3+^ and Eu^3+^ ions were chosen for their suitable energy bands that can be easily sensitized by common organic ligands, well‐separated emission bands, and the possible energy transfer between Eu^3+^ and Tb^3+^ ions. In this particular MOF matrix, the vibrations of the pendant phenyl rings are highly sensitive to temperature changes, which significantly affects the non‐radiative transition of the AIEgen ligand and the subsequent energy transfer from the ligand to the emissive Ln^3+^ ions. As a result, the mixed LnMOF exhibits high sensitivity as a ratiometric thermometer. The mixed LnMOF was successfully synthesized at the nanoscale for intracellular temperature sensing. After further modification with DSPE‐PEG2000, the nanothermometer can be easily introduced into living cells, realizing the intracellular temperature mapping in HeLa cells and L929 cells.

## 2 Results and Discussion

### 2.1 Preparation and Characterization of TbTPDB and Tb_1‐_ *_x_* Eu*_x_* TPDB

AIEgen ligand H_2_TPDB (2′,5′‐diphenyl‐[1,1′:4′,1″‐terphenyl]‐4,4″‐dicarboxylic acid) is designed as a half‐substituted tetraphenylbenzene derivative, in which only two phenyl rings at the para positions are functionalized with carboxylate groups, leaving the other two unmodified. In this case, the two carboxylated benzene rings can participate in coordination to incorporate H_2_TPDB into the LnMOF matrix. Meanwhile, the two unsubstituted phenyl rings can freely rotate or vibrate, serving as AIE motifs. Their intramolecular motions can be modulated by external stimuli, thereby achieving stimulus‐responsive luminescence. TbTPDB was obtained through a solvothermal method by the reaction of Tb(NO_3_)_3_·6H_2_O and H_2_TPDB. Single crystal X‐ray diffraction (SCXRD) shows that TbTPDB crystallizes in the monoclinic space group *I*2/a and is isostructural with EuTPDB in our previous work^[^ ^57^ ^]^ (Table S1, Supporting Information). In TbTPDB, each Tb^3+^ ion is eight‐coordinated and linked with four TPDB^2−^ ligands, forming a three‐dimensional structure with one‐dimensional rhombus channels (Figure S1, Supporting Information). The channels provide sufficient space for the intramolecular motions of the pendant phenyl rings. The closest centroid‐centroid distance between two dangling phenyl rings of the neighboring ligands is 5.42 Å. As the diameter of the phenyls is 4.00 Å, the dangling phenyl rings can freely rotate/vibrate to a certain extent^[^ ^57^ ^]^ (Figure 1b). Therefore, the intramolecular motions of these phenyl rings can be more readily modulated by temperature compared to those in conventional MOFs. Powder X‐ray diffraction (PXRD) analysis confirms the high purity of TbTPDB (Figure 1c). When Tb(NO_3_)_3_·6H_2_O and Eu(NO_3_)_3_·6H_2_O with different molar ratios were employed as starting materials, the isostructural mixed lanthanide MOFs Tb_1‐_ *_x_* Eu*_x_* TPDB (*x* = 0.005, 0.01, 0.02, and 0.03) were obtained. The PXRD patterns of Tb_1‐_ *_x_* Eu*_x_* TPDB are in accordance with the simulated PXRD patterns of EuTPDB and TbTPDB, demonstrating that all Tb_1‐_ *_x_* Eu*_x_* TPDB are isostructural to their parent LnMOFs. The final molar ratios of Tb^3+^/Eu^3+^ ions were determined by inductively coupled plasma (ICP) spectroscopy and the results are listed in Table S2 (Supporting Information). The thermogravimetric analyses (TGA) curve shows that the mixed MOFs have a high thermal stability with a decomposition temperature of 320 °C (Figure S2, Supporting Information). Tb_0.98_Eu_0.02_TPDB shows two degradation stages. The first degradation stage is between 70 and 400 °C with a weight loss of 18.9 wt.%, which may be attributed to the loss of the solvent molecules (H_2_O and DMF, 20.9 wt.% for the theoretical). As the temperature further increases, the Tb_0.98_Eu_0.02_TPDB decomposes in the second degradation stage (500–600 °C). Thermogravimetric analysis indicates that the Tb_0.98_Eu_0.02_TPDB shows thermal stability in the physiological temperature range.

### 2.2 Luminescence Properties of Tb_1‐_ *_x_* Eu*_x_* TPDB

The optical properties of TbTPDB and Tb_1‐_ *_x_* Eu*_x_* TPDB series were first investigated by steady‐state photoluminescence spectra at room temperature (Figures S3–S5, Supporting Information). When excited at 338 nm, both TbTPDB and EuTPDB exhibit the characteristic lanthanide luminescence with sharp and well‐separated emission bands. The emission spectrum of TbTPDB consists of four sharp emission lines at 488, 542, 588, and 621 nm, which originate from the transitions from the ^5^D_4_ level to the ^7^F_J_ levels (J = 6, 5, 4, and 3). Amongst them, the ^5^D_4_ – ^7^F_2_ line at 542 nm is the prominent one and is responsible for the green emission. EuTPDB displays sharp emission lines at 592, 615, 652, and 698 nm from the ^5^D_0_ → ^7^F_J_ (J = 1, 2, 3, and 4) transitions of Eu^3+^ ions and the ^5^D_0_ → ^7^F_2_ line at 615 nm dominates the emission spectrum. The emission band of the ligand was nearly unobserved in the luminescence spectra of the LnMOFs, indicating that the ligand can effectively sensitize both Tb^3+^ and Eu^3+^ ions through the antenna effect. The UV–vis absorption spectra of Tb_1‐_ *_x_* Eu*_x_* TPDB confirm the antenna effect in the Tb_1‐_ *_x_* Eu*_x_* TPDB (Figure S6, Supporting Information). All Tb_1‐_ *_x_* Eu*_x_* TPDB show the characteristic transitions of both Tb^3+^ and Eu^3+^ when excited at 338 nm (Figure S7, Supporting Information). As the concentration of Eu^3+^ ions increases from 0.005 to 0.03, the intensity of Eu^3+^ emission gradually becomes stronger. In Tb_0.98_Eu_0.02_TPDB, the emission intensities of the ^5^D_0_ → ^7^F_2_ transition of Eu^3+^ and the ^5^D_4_ – ^7^F_2_ transition of Tb^3+^ are comparable. The absolute quantum yields (QYs) of TbTPDB, EuTPDB, and Tb_1‐_ *_x_* Eu*_x_* TPDB at room temperature were measured and are listed in Table S3 (Supporting Information). The QYs of TbTPDB and the Tb_1‐_ *_x_* Eu*_x_* TPDB series fall in the range of 41.52–55.39%. In the mixed lanthanide MOFs, the quantum yield decreases as the concentration of Eu^3+^ ions increases.

**Figure 2:**
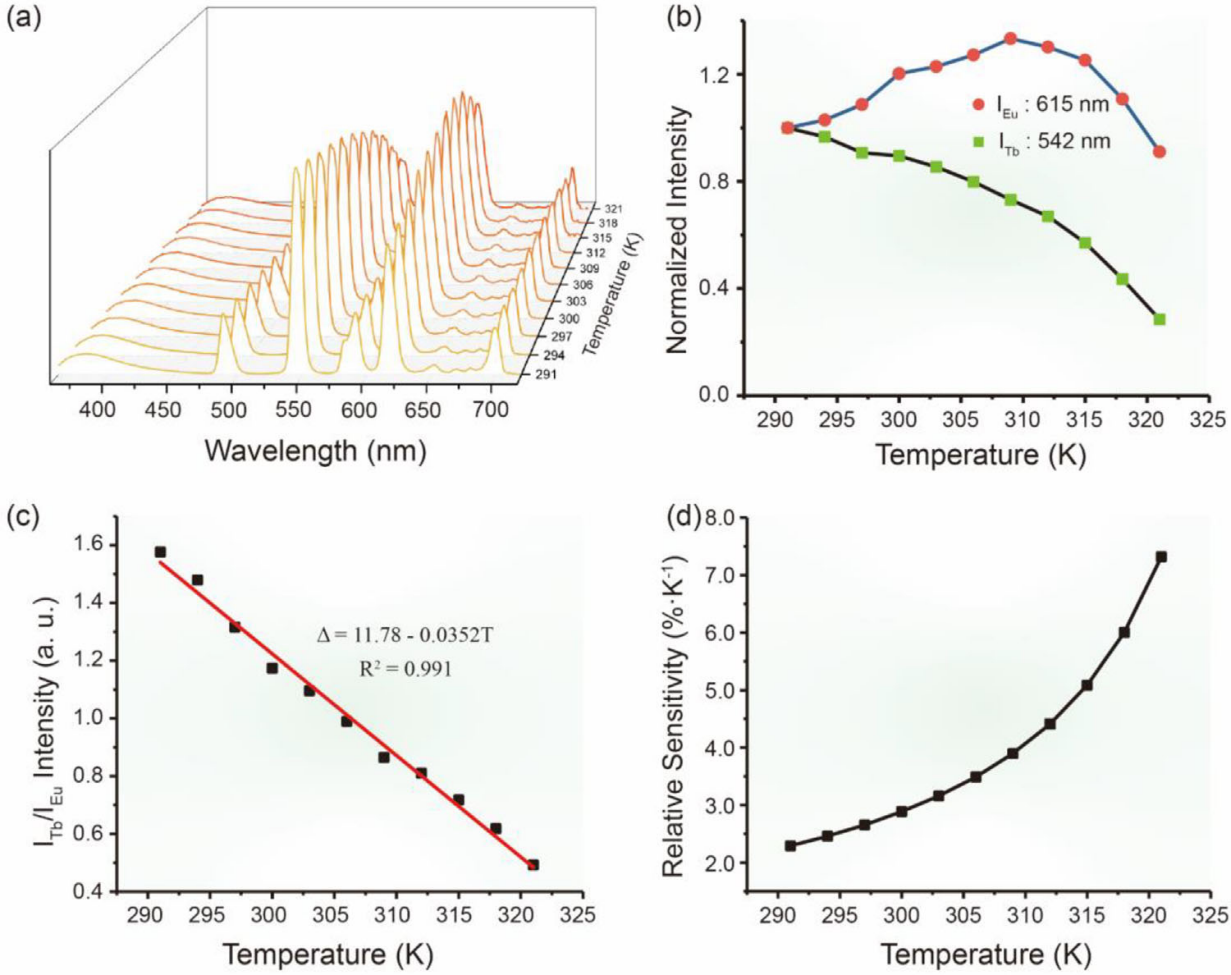
a) Emission spectra of Tb_0.98_Eu_0.02_TPDB recorded from 291 to 321 K (λ_ex_ = 338 nm). b) Temperature‐dependence of the normalized intensity of 542 nm for Tb^3+^ (^5^D_4_ → ^7^F_5_) and 615 nm for Eu^3+^ (^5^D_0_ → ^7^F_2_) transitions for Tb_0.98_Eu_0.02_TPDB. c) Temperature‐dependent intensity ratio of 542 nm (Tb^3+^) to 615 nm (Eu^3+^) and the fitted curve for Tb_0.98_Eu_0.02_TPDB. d) The relative sensitivity (S_r_) for Tb_0.98_Eu_0.02_TPDB at different temperatures.

Temperature‐dependent photoluminescence properties of Tb_1‐_ *_x_* Eu*_x_* TPDB were investigated. The temperature‐dependent emission spectra of Tb_1‐_ *_x_* Eu*_x_* TPDB from 291 to 321 K are shown in **Figure** **2**a and Figures S8–S10 (Supporting Information). The temperature‐dependent luminescence behaviors of the Tb_1‐_ *_x_* Eu*_x_* TPDB series show slight variations with different concentration ratios of Eu^3+^ and Tb^3+^ ions. In Tb_0.97_Eu_0.03_TPDB, both the intensities of 542 nm (^5^D_4_ → ^7^F_5_, ascribed to Tb^3+^) and 615 nm (^5^D_0_ → ^7^F_2_, ascribed to Eu^3+^) decrease monotonically as temperature increased (Figure S11, Supporting Information). In contrast, the intensities of Eu^3+^ emissions at 615 nm in Tb_0.98_Eu_0.02_TPDB, Tb_0.99_Eu_0.01_TPDB, and Tb_0.995_Eu_0.005_TPDB demonstrate an enhancement with rising temperature at first, followed by a subsequent decrease at higher temperatures (Figure 2b; Figures S12 and S13, Supporting Information). In all Tb_1−x_Eu_x_TPDB compounds, the intensity ratio of Tb^3+^ and Eu^3+^ emissions exhibits a strong linear dependence on temperature between 291 and 321 K, demonstrating their potential as highly sensitive ratiometric thermometers for physiological applications. Taking Tb_0.98_Eu_0.02_TPDB as an example, a linear correlation exists between absolute temperature and the intensity ratio *Δ* (*Δ* = I_Tb_/I_Eu_) from 291 to 321 K, where I_Tb_ and I_Eu_ correspond to the D_4_ → ^7^F_5_ (Tb^3+^ at 542 nm) and the ^5^D_0_ → ^7^F_2_ (Eu^3+^ at 615 nm) transitions, respectively. The relationship can be expressed as the equation *Δ* = 11.78–0.0352T (R^2^ = 0.991) (Figure 2c). The relative sensitivity (S_r_), a parameter for evaluating ratiometric thermometer performance, is defined as:

(1) $$\mathrm{Sr}=\frac{\left(\partial \Delta /\partial T\right)}{\Delta }$$

**Figure 3:**
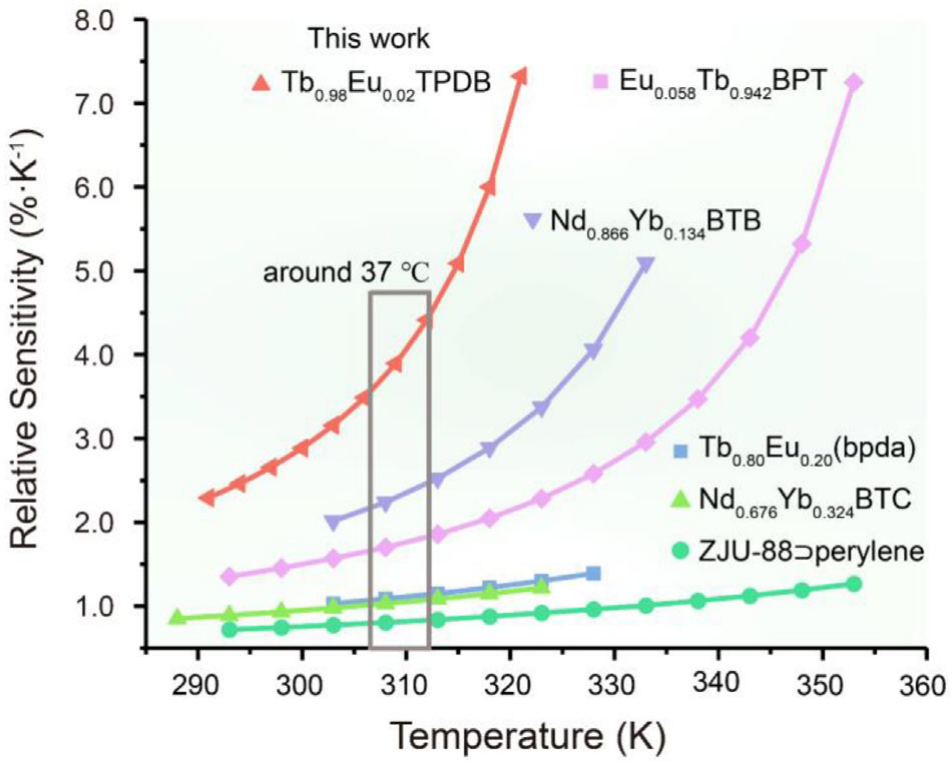
Comparison of the sensitivities of MOF‐based ratiometric thermometers in the physiological temperature range.

Following this definition, the relative sensitivities (S_r_) of Tb_1−x_Eu_x_TPDB can be determined, as shown in Figure S14 (Supporting Information). The relative sensitivity of Tb_0.98_Eu_0.02_TPDB remains above 2.29% K^−1^ in the temperature range from 291 to 321 K, and the maximum relative sensitivity (S_m_) of Tb_0.98_Eu_0.02_TPDB is 7.32% K^−1^ at 321 K (Figure 2d). The S_m_ is at a high level among MOF‐based linear ratiometric thermometers in all the temperature ranges (Table S4, Supporting Information). It is worth mentioning that the S_m_ of Tb_0.98_Eu_0.02_TPDB stands for the highest value in the physiological temperature range, and the S_r_ is remarkably higher than those for previously reported MOF‐based thermometers ≈310 K (37 °C) (**Figure** **3**). The lifetimes of Tb^3+^ (542 nm) and Eu^3+^ (615 nm) emissions in Tb_0.98_Eu_0.02_TPDB were monitored at different temperatures and are listed in Table S5 and Figures S15 and S16 (Supporting Information). Specifically, the lifetime of Tb^3+^ decreases from 447.13 µs at 291 K to 259.44 µs at 321 K, and the lifetime of Eu^3+^ decays with fluctuations from 847.24 µs at 291 K to 693.62 µs at 321 K (Figure S17, Supporting Information). The energy transfer from Tb^3+^ to Eu^3+^ can be elucidated by the temperature‐dependent lifetimes of TbTPDB (Figure S18 and Table S6, Supporting Information) and Tb_0.98_Eu_0.02_TPDB (Figure S19, Supporting Information). The energy transfer efficiency (η_ET_) from Tb^3+^ to Eu^3+^ was estimated from the equation:

(2) $$\eta_{ET}=1-\;\frac{\tau_{1}}{\tau_{2}}$$

where τ_1_ and τ_2_ are the luminescence lifetimes of Tb^3+^ (542 nm) in Tb_0.98_Eu_0.02_TPDB and TbTPDB, respectively. The value of η_ET_ is ≈59.5% at 291 K, and increases to 73.8% at 321 K, indicating that energy is transferred efficiently from Tb^3+^ to Eu^3+^ in Tb_0.98_Eu_0.02_TPDB. The temperature response of the fluorescence intensity ratio is reversible in repetitive heating‐cooling cycles (Figure S20, Supporting Information). After five thermal cycles, photoluminescence intensity shows no degradation (Figure S21, Supporting Information), and there are no obvious changes in PXRD patterns of Tb_0.98_Eu_0.02_TPDB (Figure S22, Supporting Information). The Commission Internationale d'Eclairage (CIE) chromaticity diagram based on the temperature‐dependent luminescence spectra shows that the luminescence color of the mixed lanthanide MOFs changes with the variation of the temperature (Figure S23, Supporting Information). This characteristic is beneficial for their application in temperature imaging. For Tb_0.98_Eu_0.02_TPDB, the emission color changed from yellow‐green to orange as the temperature increased from 291 to 321 K.

There are several energy transfer pathways in Tb/Eu mixed LnMOFs: ligand‐to‐Ln^3+^ (including ligand → Tb^3+^ and ligand → Eu^3+^) sensitization via the antenna effect, and efficient Tb^3+^ → Eu^3+^ energy transfer within the lanthanide framework (Figure S24, Supporting Information). Temperature‐dependent photoluminescence of the pure organic ligand (H_2_TPDB) shows that the emission intensity of the ligand decreases slightly to ≈96.6% as the temperature rises from 291 to 321 K (Figure S25, Supporting Information). Temperature‐dependent photoluminescence of GdTPDB was recorded to investigate the rotation/vibration of the benzene rings in the LnMOFs (Figure S26, Supporting Information). The luminescence intensity of GdTPDB drops to ≈69.5% in this temperature range. This discrepancy indicates that the LnMOFs provide a unique matrix where the rotation/vibration of the benzene rings are more sensitive to temperature variations. The in situ FT‐IR of Tb_0.98_Eu_0.02_TPDB also shows that the vibrations of phenyl rings become more active when temperature increases from 291 to 321 K (Figure S27, Supporting Information), evidenced by the significant intensification of the υ(C═C) band at ≈1600^−1^ cm and the υ(C─H) band at ≈3050^−1^. The observation suggests that the probability of non‐radiative transitions of the ligand increases as the temperature rises. Therefore, less energy is transferred from the ligands to the Tb^3+^ and Eu^3+^ ions through the antenna effect in the heating process. Given the AIE characteristics of the ligand, we propose that the energy loss caused by non‐radiative transitions may be more pronounced in Tb_0.98_Eu_0.02_TPDB, compared to the LnMOFs based on conventional organic ligands. Temperature‐dependent lifetimes of TbTPDB and Tb_0.98_Eu_0.02_TPDB demonstrate that the efficiency of energy transfer from Tb^3+^ to Eu^3+^ displays a significant enhancement with increasing temperature. Based on the above experiment results, an analysis of the photophysical processes of Tb_0.98_Eu_0.02_TPDB was carried out. For Tb^3+^, as the temperature increases, energy transfer from the ligand decreases while that to Eu^3+^ increases, resulting in a dramatic decrease in green emission. For Eu^3+^, the energy transfer from the ligand decreases as the temperature rises. However, this energy loss may be offset by the increased efficiency of energy transfer from Tb^3+^ to Eu^3+^. Thus, the red emission of Eu^3+^ even shows an enhancement in a certain temperature range. The opposite thermal response trends of the two emissions lead to the emission color change and a high S_m_ value in Tb_0.98_Eu_0.02_TPDB, making it a highly sensitive thermometer for in cellular temperature mapping.

### 2.3 Preparation of Tb_0.98_Eu_0.02_TPDB in Nanoscale

**Figure 4:**
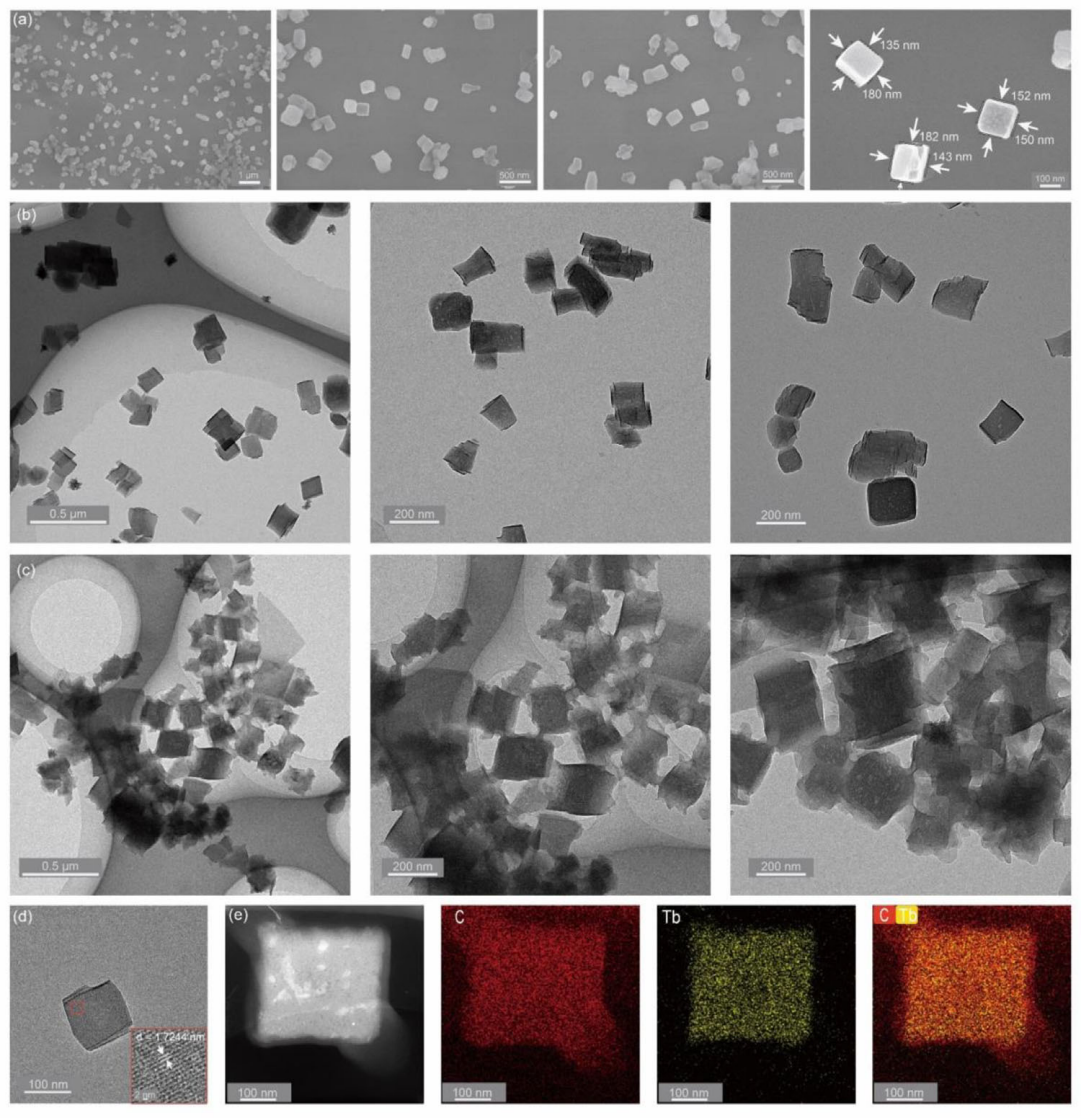
a) SEM images of Tb_0.98_Eu_0.02_TPDB NPs. b) TEM images of Tb_0.98_Eu_0.02_TPDB NPs. c) TEM images of Tb_0.98_Eu_0.02_TPDB@DSPE‐PEG2000. d) HRTEM image of Tb_0.98_Eu_0.02_TPDB NPs. e) Elemental mapping of C and Tb in Tb_0.98_Eu_0.02_TPDB@DSPE‐PEG2000.

The high sensitivity and obvious emission color change of Tb_0.98_Eu_0.02_TPDB within the physiological temperature range encourage us to explore its applicability for temperature detection in biological systems. MOF crystals obtained via solvothermal methods typically have sizes ranging from tens to hundreds of micrometers, which hinders the application of high spatial resolution temperature sensing. Thus, to achieve biological temperature imaging, it is necessary to synthesize MOF crystals at the nanoscale. Nanoparticles of Tb_0.98_Eu_0.02_TPDB were obtained through a bottom‐up hot‐injection method using polyvinylpyrrolidone (PVP) as a surfactant, denoted as Tb_0.98_Eu_0.02_TPDB NPs. PXRD patterns (Figure S28, Supporting Information) show that all peaks of Tb_0.98_Eu_0.02_TPDB NPs match well with those of the crystalline Tb_0.98_Eu_0.02_TPDB samples, indicating the structural integrity and high purity of Tb_0.98_Eu_0.02_TPDB NPs. The peaks for the (0, 0, 2) crystal plane, as well as the related faces (0, 0, 4) and (0, 0, 6), intensified due to the preferred orientation of Tb_0.98_Eu_0.02_TPDB NPs.^[^ ^58^, ^59^, ^60^ ^]^ Scanning electron microscope (SEM) images show that the Tb_0.98_Eu_0.02_TPDB NPs exhibit a cuboidal morphology with an average length of ≈150 nm (**Figure** **4**a). Transmission electron microscopy (TEM) images further confirm that Tb_0.98_Eu_0.02_TPDB NPs are rectangular solid samples with sizes in the range of 150–200 nm (Figure 4b).

To ensure good biocompatibility of Tb_0.98_Eu_0.02_TPDB NPs, 1,2‐distearoyl‐sn‐glycero‐3‐phosphoethanolamine‐N‐[methoxy(polyethylene glycol)‐2000] (DSPE‑PEG2000), an amphiphilic phospholipid, was used to modify the nanoparticle surface via the Bangham method,^[^ ^61^, ^62^ ^]^ yielding Tb_0.98_Eu_0.02_TPDB@DSPE‐PEG2000. TEM images reveal that the DSPE‐PEG2000‐modified samples exhibit an increased average size of ≈200 nm (Figure 4c), showing a thin amorphous shell surrounding the core of Tb_0.98_Eu_0.02_TPDB. The high‐resolution TEM (HRTEM) image (Figure 4d) shows that the Tb_0.98_Eu_0.02_TPDB NPs have distinct lattice fringes with a spacing of 1.7244 nm ascribed to the (0, 0, 2) crystal plane (Figure S24, Supporting Information). The elemental mapping images illustrate that the Tb element is uniformly distributed in the core of Tb_0.98_Eu_0.02_TPDB@DSPE‐PEG2000, while the C element is distributed in both core and shell, verifying the success of surface modification. The zeta potential was measured to be ≈−10 mV (Figure S29, Supporting Information). Dynamic light scattering (DLS) measurements indicated a hydrodynamic diameter of 184 nm in aqueous solution (Figure S30, Supporting Information), consistent with the results obtained from SEM and TEM images. Meanwhile, the PXRD patterns show that the structure of Tb_0.98_Eu_0.02_TPDB still maintains after modification with DSPE‐PEG2000 (Figure S28, Supporting Information). Tb_0.98_Eu_0.02_TPDB@DSPE‐PEG2000 shows a high stability toward air and water. When the core‐shell nanoparticles were placed in the open air for one week or dispersed in aqueous solutions at different pH values (pH 6 or 8) for 24 h, no obvious changes were observed in the PXRD patterns of the treated samples (Figures S31 and S32, Supporting Information). In summary, nanosized Tb_0.98_Eu_0.02_TPDB was successfully prepared, and DSPE‐PEG2000 can be modified on the surface of the NPs, obtaining a stable composite suitable for intracellular thermometry.

### 2.4 Confocal Laser Scanning Microscopy Imaging

The cytotoxicity of the thermometer was evaluated by the methyl thiazolyl tetrazolium (MTT) assay in the human cervical carcinoma cell line HeLa (Details in Experimental Section, Supporting Information). As shown in Figure S33 (Supporting Information), when the cells were incubated with unmodified Tb_0.98_Eu_0.02_TPDB NPs for 24 h, the viability of HeLa cells decreased to ≈46.7% as the concentration increased from 0 to 200 µg mL^−1^. In contrast, the cells incubated with Tb_0.98_Eu_0.02_TPDB@DSPE‐PEG2000 maintained viability above 80% (Figure S34, Supporting Information). The MTT results indicate that the modified Tb_0.98_Eu_0.02_TPDB NPs have good biocompatibility and that DSPE‐PEG2000 plays an essential role in this property.

**Figure 5:**
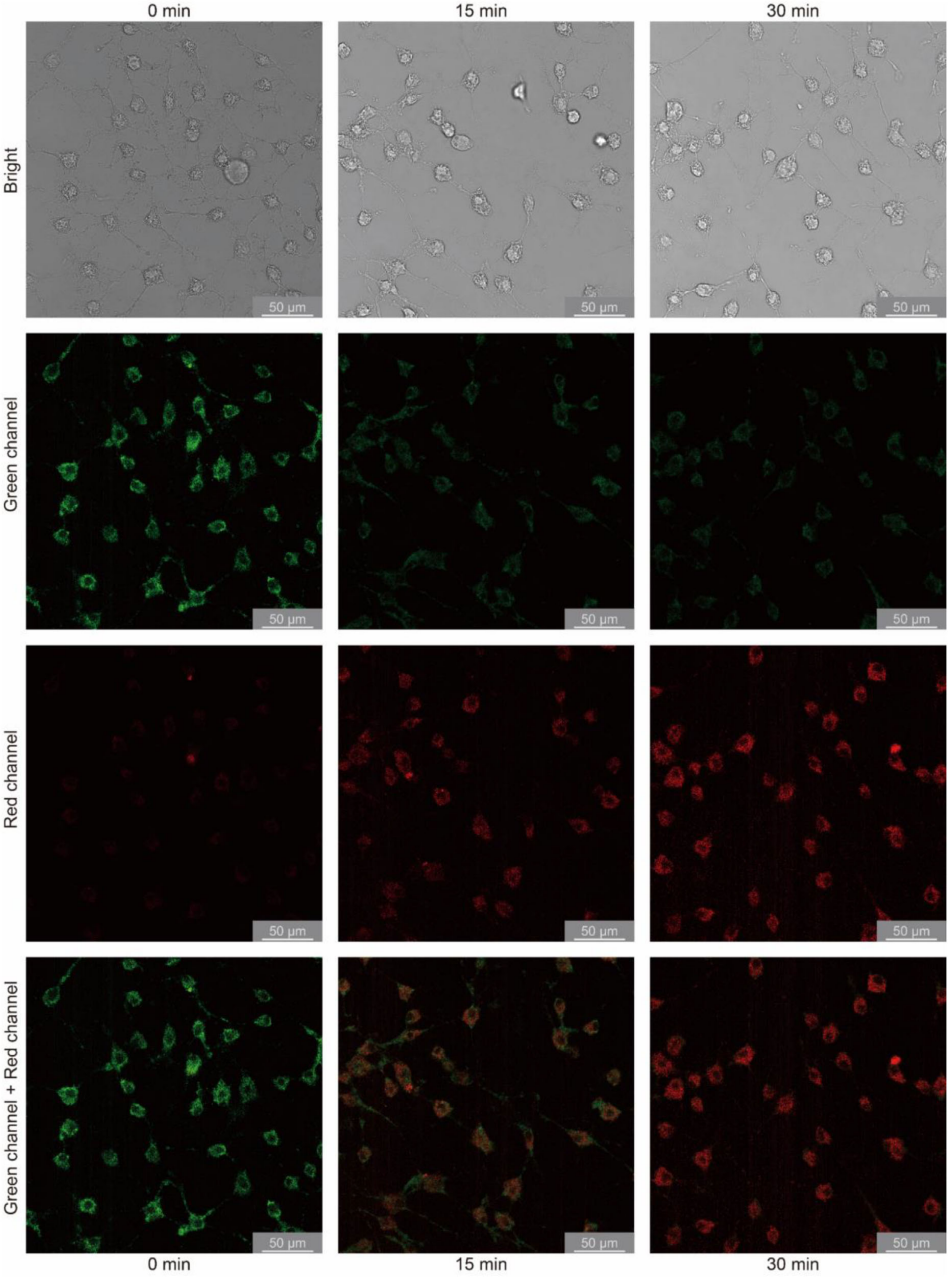
Confocal laser scanning microscopy images of L929 cells, treated with lipopolysaccharide for different times. Bright field images (the first row), green channel (the second row), red channel (the third row), green channel and red channel (the fourth row). Excitation wavelength: 405 nm.

Then, Tb_0.98_Eu_0.02_TPDB@DSPE‐PEG2000 was introduced to L929 mouse fibroblast cells or HeLa human cervical adenocarcinoma cells to test its capability for intracellular temperature detection. After incubating the cells in 300 µg mL^−1^ Tb_0.98_Eu_0.02_TPDB@DSPE‐PEG2000 solution for 2 h, confocal laser scanning microscopy (CLSM) imaging clearly demonstrated that the composite had been successfully phagocytosed by L929 cells based on the comparison of the bright field image and the fluorescence image in the green channel at 0 min (**Figure** **5**). The fluorescence in the red channel at 0 min was very weak and almost invisible. Then, lipopolysaccharide (LPS), a kind of immune activator that can cause an acute inflammatory response to increase cellular temperature, was introduced. After 15 min of lipopolysaccharide treatment in L929 cells, the fluorescence in the red channel appeared while the emission in the green channel faded as the intracellular temperature rose, accompanied by a color change from green to orange in the overlap images. Upon LPS stimulation for ≈30 min, the fluorescence in the green channel further weakened and became almost invisible as the intracellular temperature continued to increase. At this time, only the fluorescence in the red channel could be observed, and the fluorescence in the overlap images changed from green to red. The whole process was observed directly with the naked eye, showing that the composite could be used as a ratiometric luminescence thermometer for intracellular temperature mapping. The fluorescence of Tb_0.98_Eu_0.02_TPDB@DSPE‐PEG2000 showed an analogous temperature behavior in HeLa cells: the emission color changed from green to red when stimulated by LPS. The difference was that the change of the emission color was more pronounced in L929 cells (Figure S35, Supporting Information). This difference may be ascribed to the different phagocytic activities and metabolic rates between the two kinds of cells. On the whole, the MOF‐based ratiometric thermometer can monitor intracellular thermal variation by significant emission color change. To the best of our knowledge, this is the first MOF‐based ratiometric thermometer that has been successfully applied to intracellular temperature sensing.

## 3 Conclusion

In summary, by coordinating AIEgen ligands with mixed lanthanide ions, we have successfully constructed a series of mixed lanthanide MOFs Tb_1‐_ *_x_* Eu*_x_* TPDB, that can serve as highly sensitive ratiometric thermometers. Among them, Tb_0.98_Eu_0.02_TPDB exhibits a maximum relative sensitivity (S_m_) of 7.32% K^−1^ at 321 K, outperforming other reported ratiometric luminescent MOFs. To meet the requirements of biological temperature imaging, this highly sensitive thermometer was synthesized at a nanoscale size of ≈150 nm. The NPs were then surface‐modified with DSPE‐PEG2000 to obtain good biocompatibility and low biotoxicity. Tb_0.98_Eu_0.02_TPDB@DSPE‐PEG2000 was subsequently introduced to L929 cells or HeLa cells. Upon stimulation with LPS to induce an increase in intracellular temperature, the process could be directly visualized with the naked eye as the fluorescence in the overlap images turns from green to red. This study demonstrates a promising strategy for developing highly sensitive MOF‐based ratiometric nanothermometers toward intracellular temperature imaging.

## Conflict of Interest

The authors declare no conflict of interest.

## Supporting information



Supporting Information

Supporting Information

## Data Availability

CCDC 2453248 contains the supplementary crystallographic data for this paper. These data can be obtained free of charge from The Cambridge Crystallographic Data Centre via www.ccdc.cam.ac.uk/data_request/cif.
