# Supplementary material for: A Sensitive Ratiometric Thermometer Constructed by AIEgen‐Based Mixed Lanthanide MOF for Intracellular Temperature Mapping
Source: Adv Sci (Weinh). 2025 Aug 12;12(41):e10147. doi: 10.1002/advs.202510147 (PMC12591144; doi:10.1002/advs.202510147)
Supplement: Supplementary file 1 — Supporting Information [file ADVS-12-e10147-s001.docx]

Supporting Information

**A sensitive** **ratiometric thermometer constructed by AIEgen-based mixed** **lanthanide MOF for intracellular temperature mapping**

Zhijia Li^1, 2^, Jieping Zhang^2^, Zhiyuan Wu^2^, Hang Lei^2^, Yunfang Zhao^2^, Wenqiu Qi^2, 3^, Xue Gao^2^, Feilong Jiang^2^, Yongsheng Liu^1, 2^, Lian Chen^1, 2^*, Maochun Hong^1, 2^*

^1^Fujian Science & Technology Innovation Laboratory for Optoelectronic Information of China, Fuzhou, Fujian 350108, China

^2^State Key Laboratory of Structure Chemistry, Fujian Institute of Research on the Structure of Matter, Chinese Academy of Sciences, Fuzhou 350002, China.

^3^College of Chemistry and Materials Science, Fujian Normal University, Fuzhou, Fujian 350007, China.

E-mail: Lian Chen (cl@fjirsm.ac.cn); Maochun Hong (hmc@fjirsm.ac.cn).

**Experimental Section**

**Synthesis of TbTPDB.** A mixture of Tb(NO_3_)_3_·6H_2_O (5.7 mg, 0.0125 mmol) and 2',5'-diphenyl-[1,1':4',1''-terphenyl]-4,4''-dicarboxylic acid (H_2_TPDB) were dissolved in DMF (N,N-dimethylformamide) (2 mL), H_2_O (1 mL) and HCl (50 μL) with ultrasonic, then sealed in a 25 mL Teflon chamber within a Teflon-lined stainless steel autoclave and heated at 130 ℃ for three days^[1]^. After the mixture was slowly cooled to room temperature, colorless crystals TbTPDB were obtained, washed with DMF and MeOH, and dried in air. The yield is ca. 55.4% for TbTPDB based on the ligand. Elemental analysis (%): Calcd (%) for TbC_38_H_38_N_3_O_11_ (Mr= 871.65): C, 52.36; H, 4.40; N, 4.82; O, 20.19. Found (%): C, 51.81; H, 4.76; N, 4.33; O, 20.52.

**Synthesis of Tb_1-_*_x_*Eu*_x_*TPDB.** The mixed lanthanide MOFs Tb_1-_*_x_*Eu*_x_*TPDB (*x* = 0.005, 0.01, 0.02, 0.03) were synthesized following a similar procedure^[1]^, except that a mixture of Tb(NO_3_)_3_·6H_2_O and Eu(NO_3_)_3_·6H_2_O with different ratios was used as starting materials. The PXRD patterns indicate that mixed lanthanide MOFs are isostructural to TbTPDB. The molar ratios of Tb/Eu in these mixed lanthanide MOFs were determined by the ICP analyses (Table S2).

**Synthesis of** **Tb_0.98_Eu_0.02_TPDB** **NPs.** Tb(NO_3_)_3_·6H_2_O (111.0 mg, 0.245 mmol), Eu(NO_3_)_3_·6H_2_O (2.2 mg, 0.005 mmol), polyvinylpyrrolidone (PVP) (20.0 g), and trichloroacetic acid (TCA) (3.2 g) were mixed with the solution of DMF (N,N-dimethylformamide) (20 mL) and H_2_O (20 mL) in a two necked flask. Then, the flask was heated to 130 ℃ through oil bath heating. When the mixture was completely dissolved, 20 mL of DMF containing H_2_TPDB (117.5 mg, 0.250 mmol) was slowly injected into the reaction solution. After reaction at 130 ℃ for about 12 h, the mixture was cooled to room temperature. Using a centrifuge, the entire reaction product was collected at 12000 rpm/8 min and washed with DMF and EtOH three times each. The product was then centrifuged at 6000 rpm for 5 minutes to collect the Tb_0.98_Eu_0.02_TPDB NPs in the upper suspension.

**Synthesis of** **Tb_0.98_Eu_0.02_TPDB@DSPE-PEG2000.** Tb_0.98_Eu_0.02_TPDB NPs (2 mg) and DSPE-PEG2000 (4 mg) were mixed in EtOH (1 mL) and chloroform (2 mL) in an eggplant-shaped flask. The flask was then attached to a rotary evaporator and the organic solvent was evaporated under vacuum at a temperature of 35 ℃ to obtain a thin film. Place the eggplant-shaped under vacuum at 60 ℃ overnight. Finally, the thin film was hydrated with 6 mL H_2_O at 60 ℃ for 30 min to obtain the Tb_0.98_Eu_0.02_TPDB@DSPE-PEG2000.

**MTT assay.** The cytotoxicity was evaluated by the methyl thiazolyl tetrazolium (MTT) assay in the human cervical carcinoma cell line HeLa. The HeLa cell line was obtained from iCell Bioscience Inc. and cultured in medium containing 10% calf serum at 37 ℃ in the presence of 5% CO_2_ for 24 h. Then, the cells were incubated with fresh medium containing Tb_0.98_Eu_0.02_TPDB NPs or Tb_0.98_Eu_0.02_TPDB@DSPE-PEG2000 at different concentrations (i.e., 0, 25, 50, 100, 200, 300, 400, and 500 μg/mL) at 37 ℃ in the presence of 5% CO_2_ for 24 h. Subsequently, 100 μL of MTT solution (5 mg/mL) was added to each well of the 96-well assay plate and incubated for an additional 4 h at 37 ℃ in the presence of 5% CO_2_. Following incubation, the medium was replaced with 100 μL of dimethyl sulfoxide (DMSO) per well. Finally, the 96-well plates were gently shaken for 10 min at room temperature before measuring the absorbance at 570 nm.

**Cell Culture and Confocal Laser Scanning Microscopy Imaging.** L929 cells were cultured in Dulbecco’s modified Eagle medium (DMEM) at 37 °C in a humidified atmosphere with 5% CO_2_ for 24 h. After that, the L929 cells were rinsed thrice with sterile PBS buffer. Then, incubated in 300 μg/mL Tb_0.98_Eu_0.02_TPDB@DSPE-PEG2000 solution for 2h and rinsed thrice with sterile PBS buffer to remove the unphagocytosed NPs. The L929 cells were treated with lipopolysaccharide (20 ug/mL) and images were collected with a 405 nm excitation laser at 0 min, 15 min and 30 min.

HeLa cells were cultured in Dulbecco’s modified Eagle medium (DMEM) at 37 °C in a humidified atmosphere with 5% CO_2_ for 24 h. After that, the L929 cells were rinsed thrice with sterile PBS buffer. Then, incubated in 300 μg/mL Tb_0.98_Eu_0.02_TPDB@DSPE-PEG2000 solution for 2h and rinsed thrice with sterile PBS buffer to remove the unphagocytosed NPs. The HeLa cells were treated with lipopolysaccharide (20 ug/mL) and images were collected with a 405 nm excitation laser at 0 min, 15 min and 30 min.

**Materials.** H_2_TPDB was synthesized according to our previous report^[1]^. Ln(NO_3_)_3_·6H_2_O were purchased from Beijing HwrkChemical Technology Co., Ltd without any further purification. The solvents were purchased from Sinopharm Chemical Reagent Co., Ltd without any further purification. The other relevant chemicals were purchased from Shanghai Titan Scientific Co., Ltd without any further purification.

**Characterization*.*** All the crystal structure data of compounds were collected on an XtaLAB Synergy R diffractometer equipped with Cu-Kα radiation (λ = 1.54184 Å), using a ω scan mode at 100 K.Powder X-ray diffraction (PXRD) patterns were collected with a Rigaku MiniFlex 600 diffractometer using Cu *K*α radiation (*λ* = 1.54 Å). Elemental analyses (C, H, N and O) were performed on a German Elementary Vario EL III instrument. Thermogravimetric analyses (TGA) were recorded in the temperature range of 30 – 900 ℃, with a heating rate of 10 ℃·min^-1^ under a flowing nitrogen atmosphere on a Netzsch Model STA 449C instrument. Scanning electron microscope (SEM) images were acquired on a SU8010 SEM.Transmission electron microscopy (TEM) images were acquired on a Talos-F200X TEM. Zeta potential were obtained on advanced light scattering system (Malvern Zetasizer Ultr, Malvern). All the emission and excitation spectra in the solid state were acquired on a FLS1000 spectrofluorometer with a continuous xenon lamp.


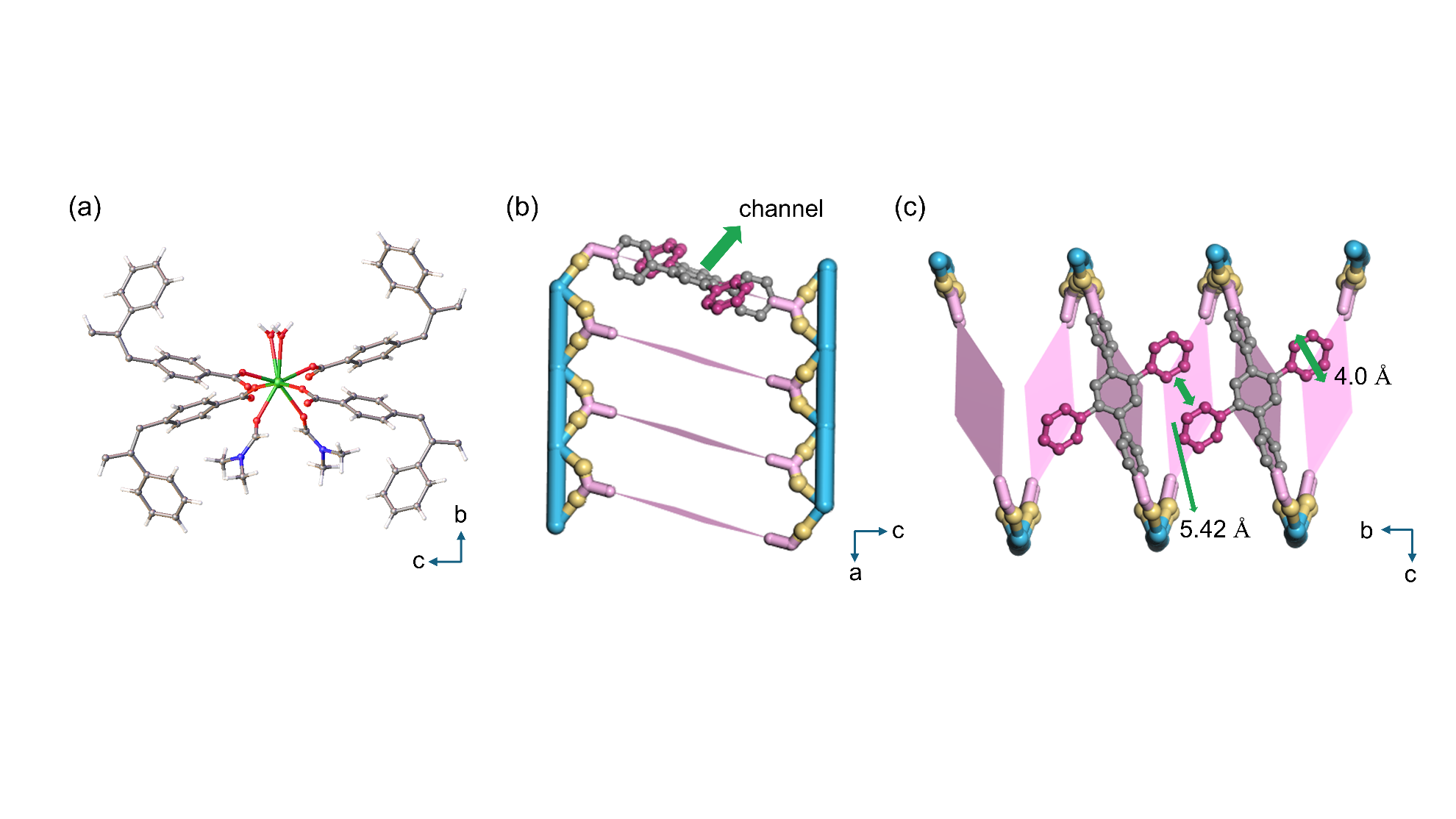


**Figure S1**. a) The coordination environment of Tb^3+^. b) The channels provide sufficient space for the intramolecular motions of the pendant phenyl rings. c) The closest distance between dangling phenyl rings on the neighboring ligands is 5.42 Å which is slightly larger than the diameter of phenyls (4.00 Å).


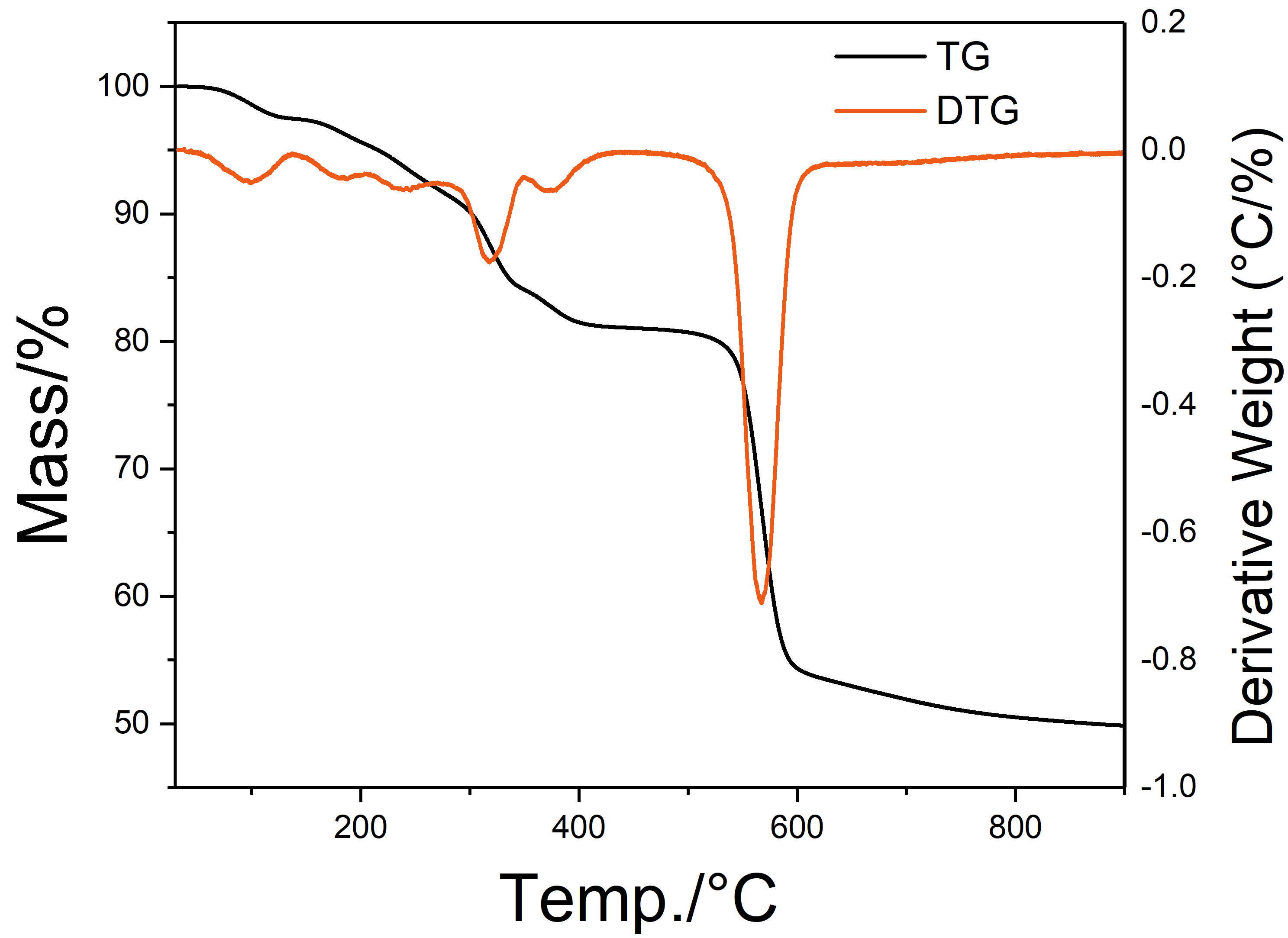


**Figure** **S2**. The thermogravimetric curve (TG) and derivative thermogravimetry (DTG) of Tb_0.98_Eu_0.02_TPDB.


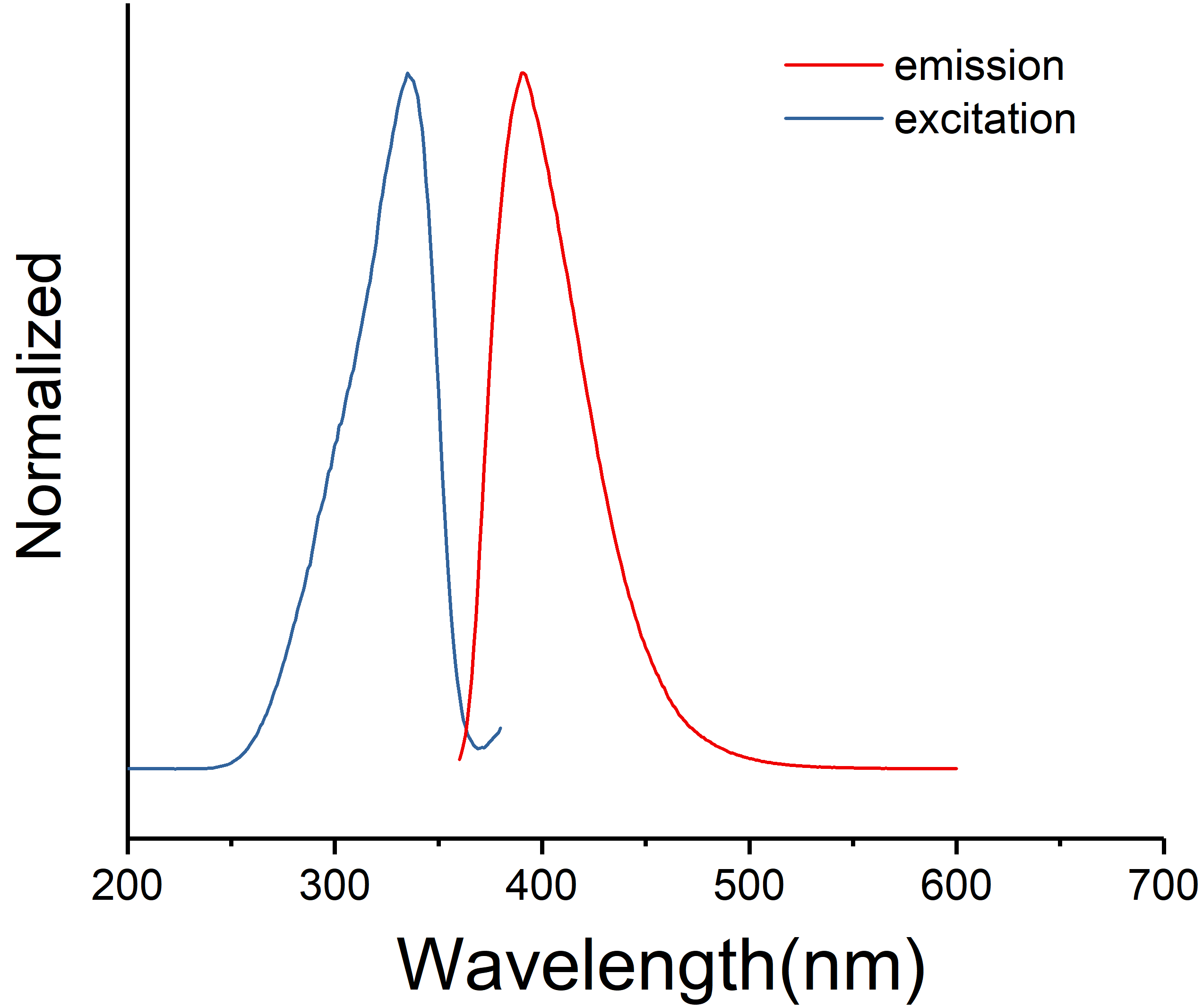


**Figure S3**. The excitation and emission spectra of H_2_TPDB at room temperature ( λ_ex_ = 338 nm, λ_em_ = 390 nm).


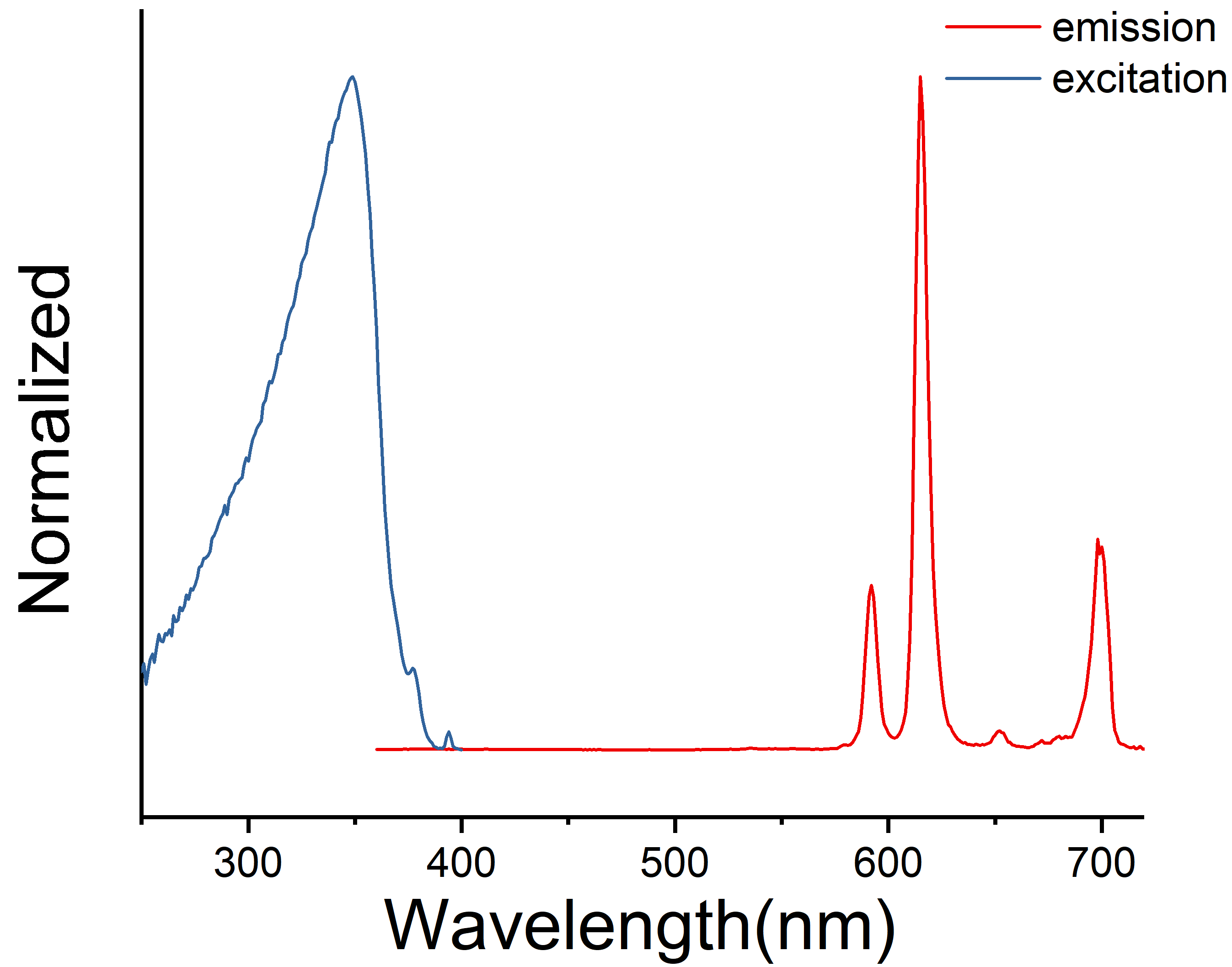


**Figure S4**. The excitation and emission spectra of EuTPDB at room temperature ( λ_ex_ = 338 nm, λ_em_ = 615 nm).


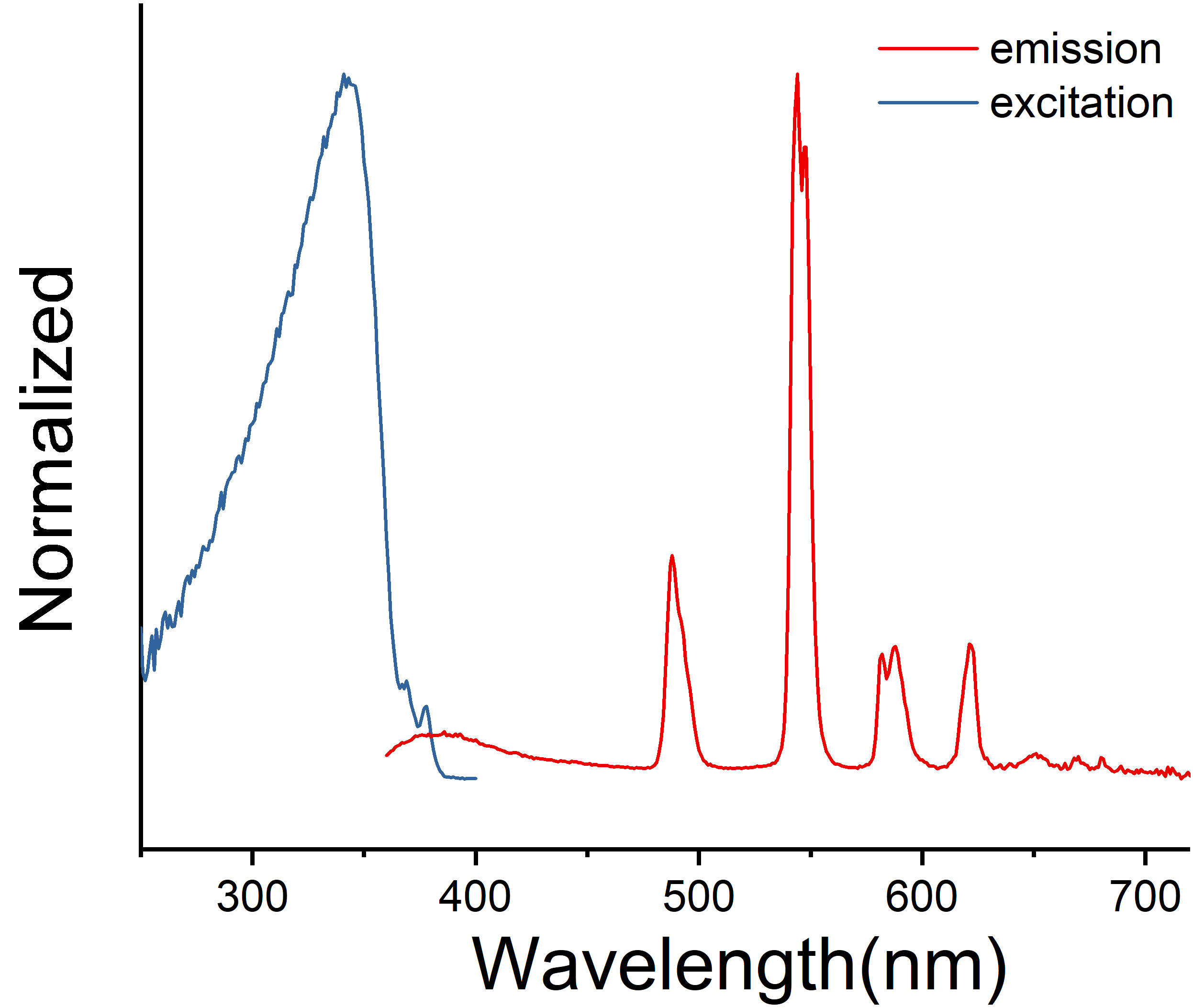


**Figure S5**. The excitation and emission spectra of TbTPDB at room temperature ( λ_ex_ = 338 nm, λ_em_ = 542 nm).


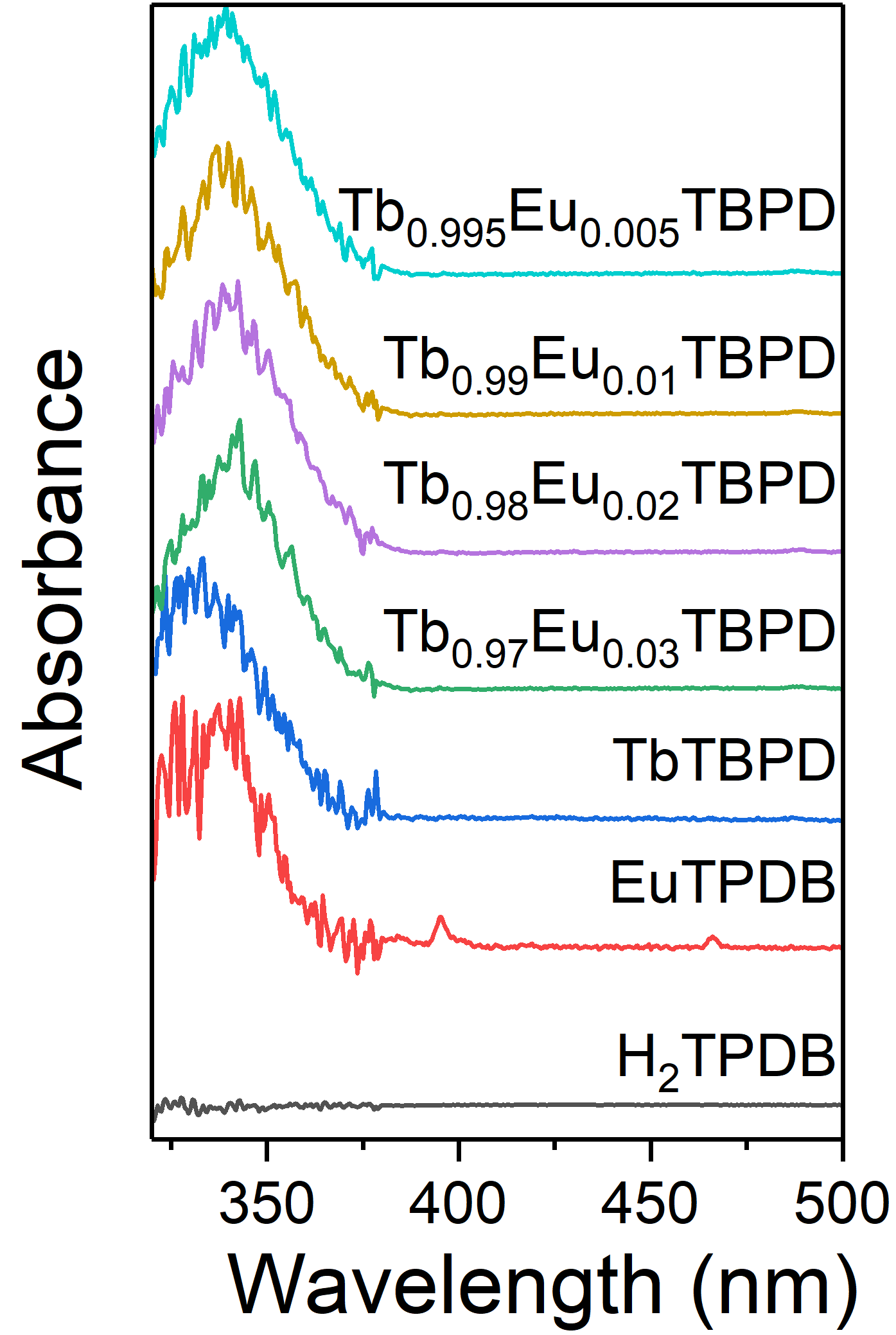


**Figure S6**. The UV-visible absorption spectra of the ligands and Tb_1-x_Eu_x_TPDB.


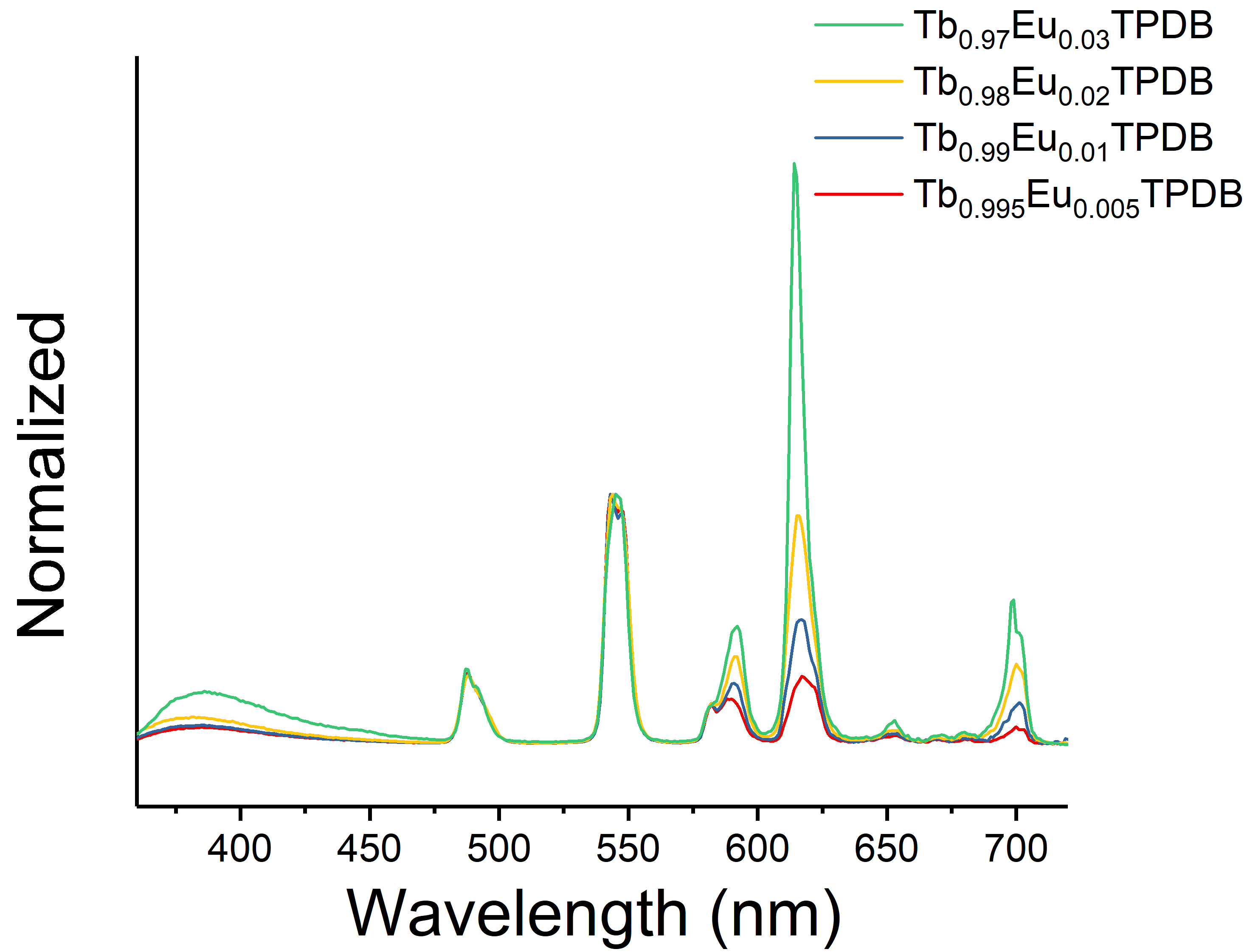


**Figure S7**. The emission spectra of Tb_1-_*_x_*Eu*_x_*TPDB (*x* = 0.005, 0.01, 0.02, 0.03) at room temperature ( λ_ex_ = 338 nm).


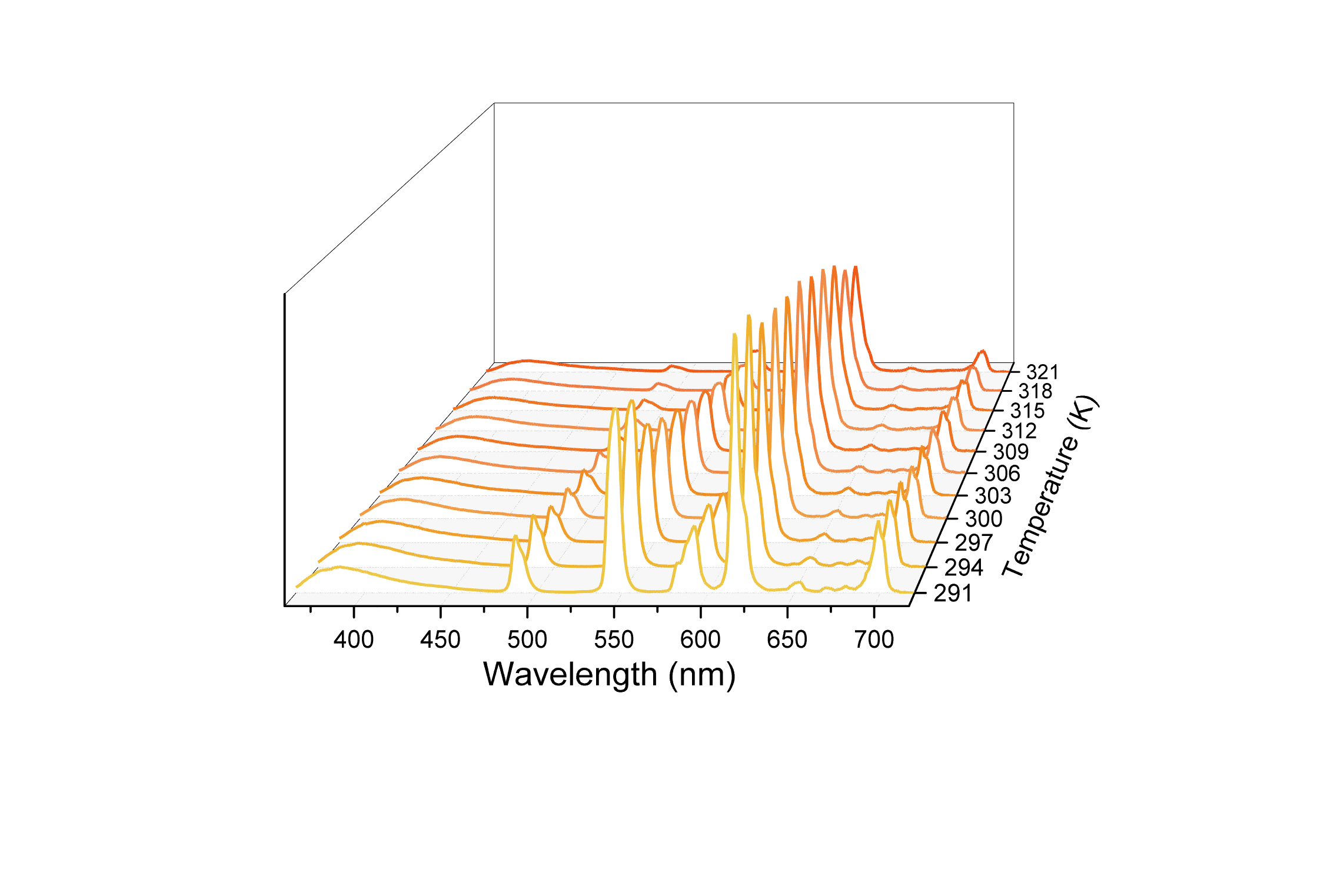


**Figure S8**. Emission spectra of Tb_0.97_Eu_0.03_TPDB recorded from 291 K to 321 K (λ_ex_ = 330 nm).


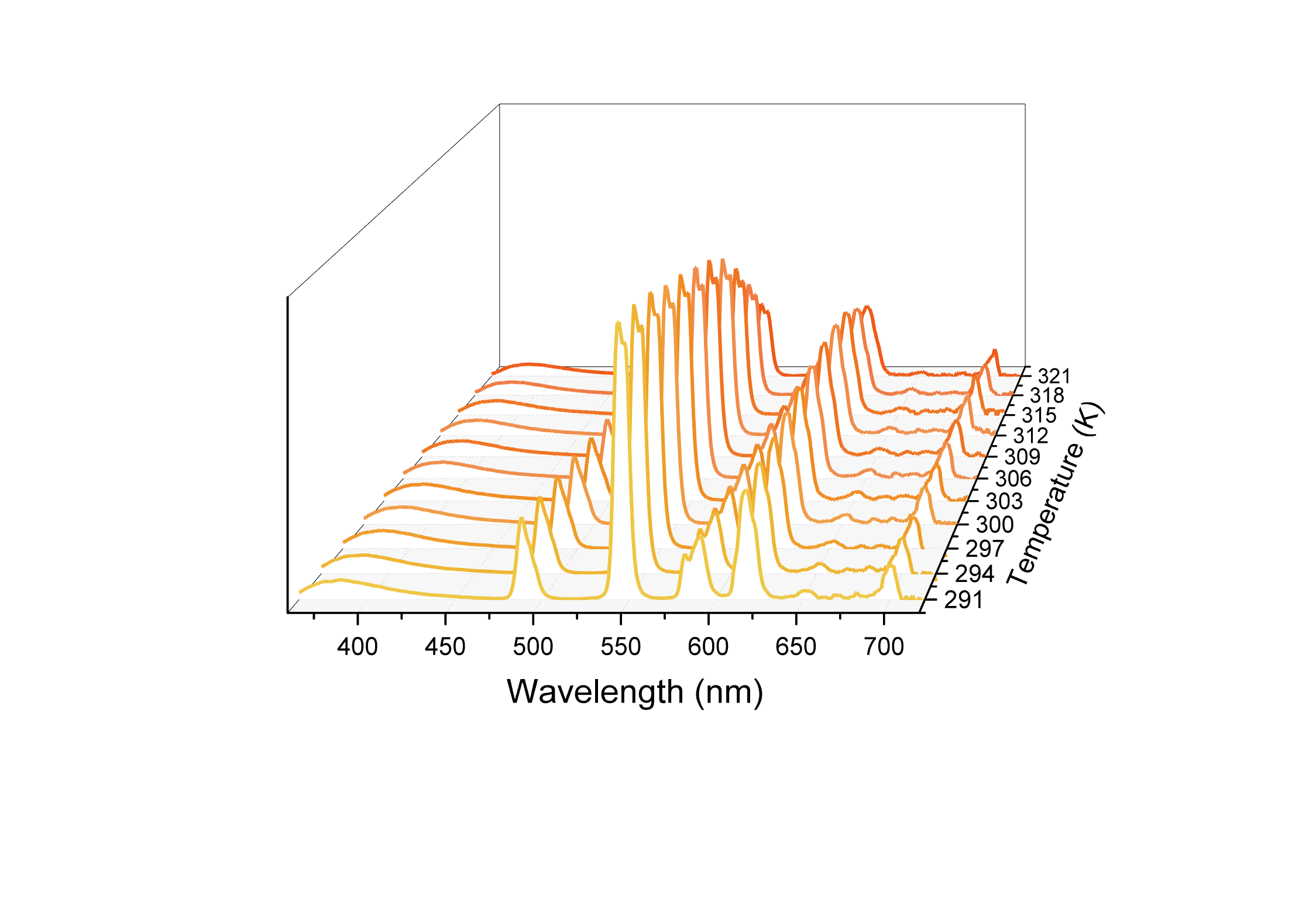


**Figure S9**. Emission spectra of Tb_0.99_Eu_0.01_TPDB recorded from 291 K to 321 K (λ_ex_ = 330 nm).


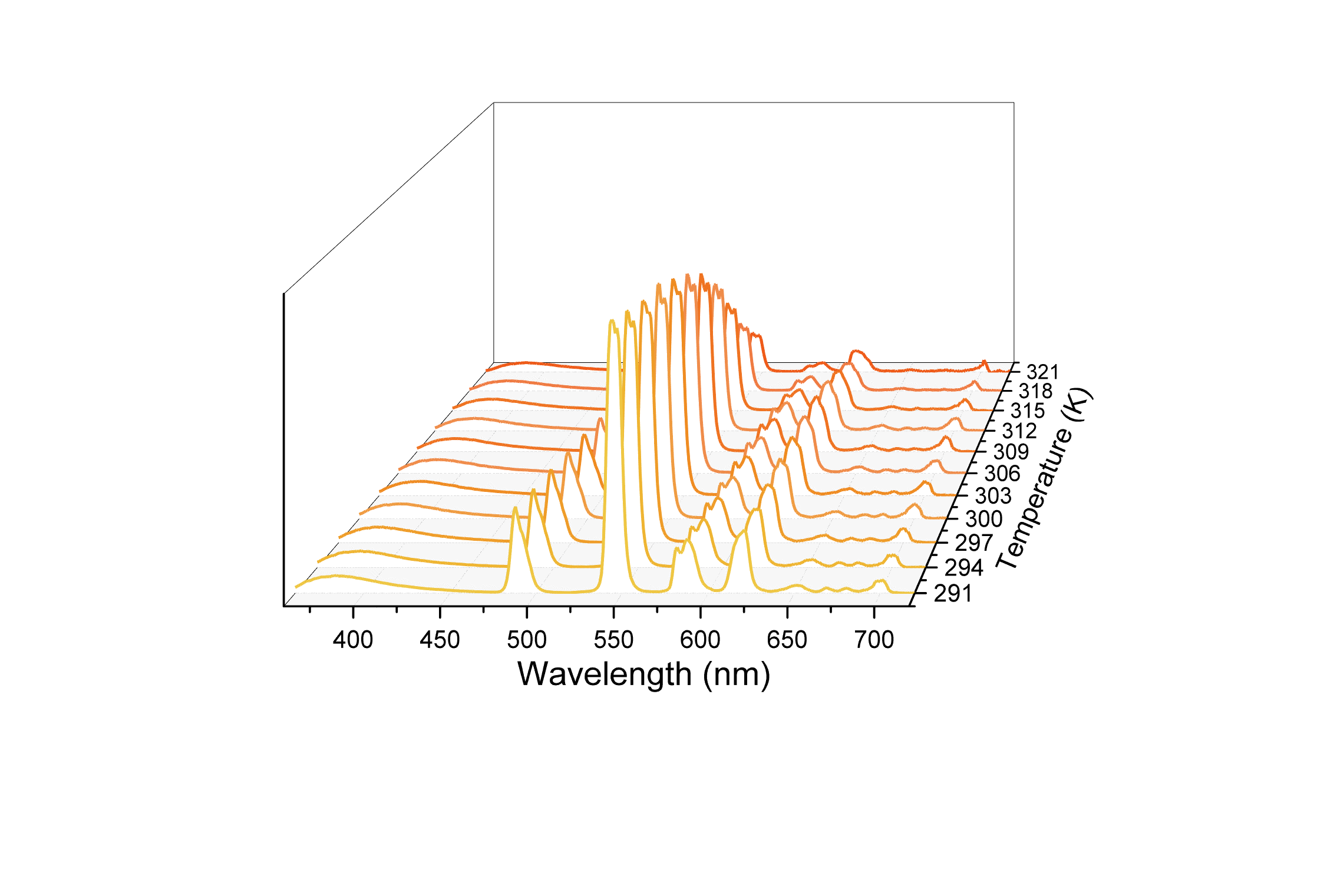


**Figure S10**. Emission spectra of Tb_0.995_Eu_0.05_TPDB recorded from 291 K to 321 K (λ_ex_ = 330 nm).


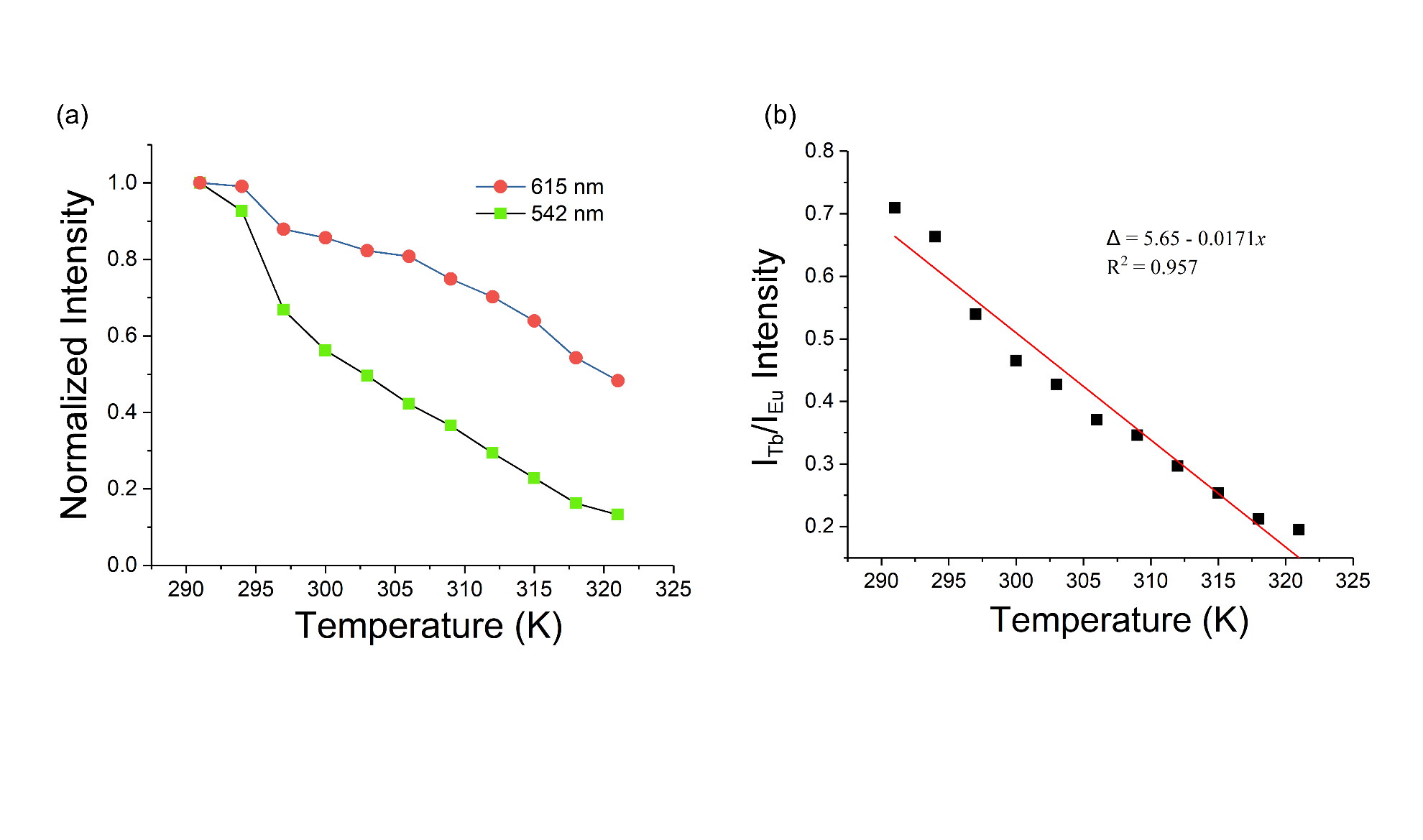


**Figure S11**. a) Temperature dependence of the normalized intensity of ^5^D_4_ → ^7^F_5_ (542 nm, Tb^3+^) and ^5^D_0_ → ^7^F_2_ (615 nm, Eu^3+^) transitions for Tb_0.97_Eu_0.03_TPDB. b) Temperature-dependent normalized intensity ratio of *I*_Tb_ (^5^D_4_ → ^7^F_5_) to *I*_Eu_ (^5^D_0_ → ^7^F_2_) and the fitted curve for Tb_0.97_Eu_0.03_TPDB.


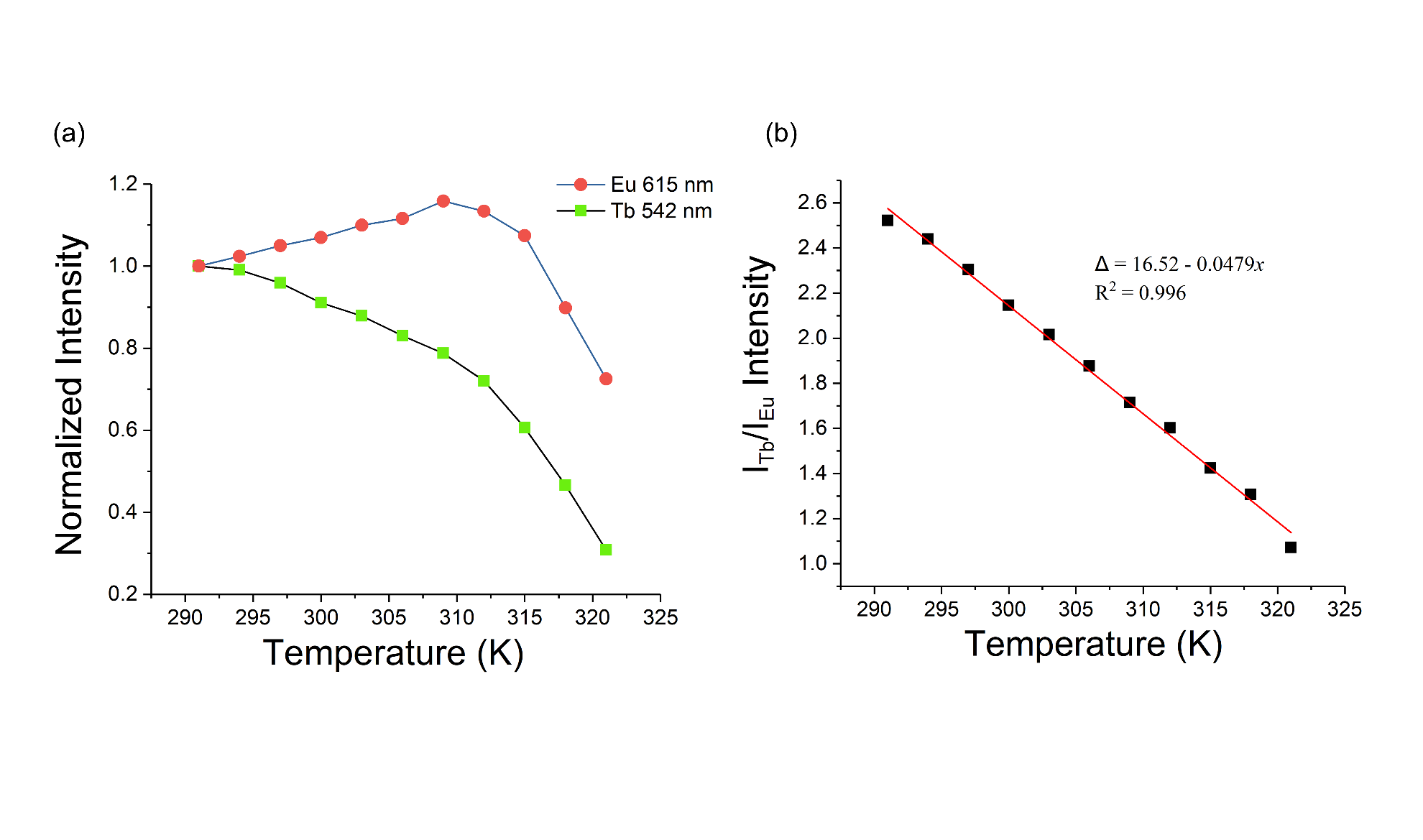


**Figure S12**. a) Temperature dependence of the normalized intensity of ^5^D_4_ → ^7^F_5_ (542 nm, Tb^3+^) and ^5^D_0_ → ^7^F_2_ (615 nm, Eu^3+^) transitions for Tb_0.99_Eu_0.01_TPDB. b) Temperature-dependent normalized intensity ratio of *I*_Tb_ (^5^D_4_ → ^7^F_5_) to *I*_Eu_ (^5^D_0_ → ^7^F_2_) and the fitted curve for Tb_0.99_Eu_0.01_TPDB.


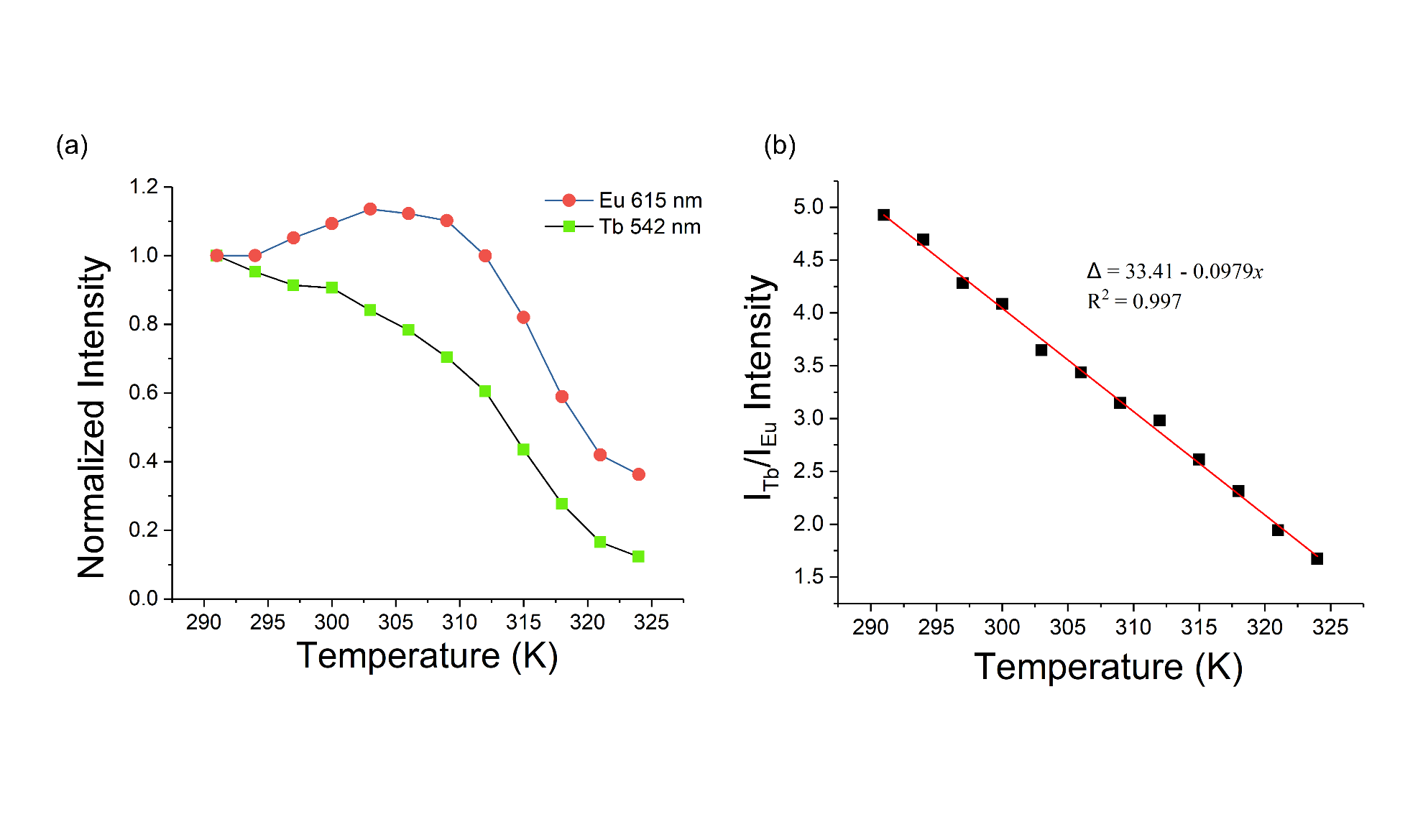


**Figure S13**. a) Temperature dependence of the normalized intensity of ^5^D_4_ → ^7^F_5_ (542 nm, Tb^3+^) and ^5^D_0_ → ^7^F_2_ (615 nm, Eu^3+^) transitions for Tb_0.995_Eu_0.05_TPDB. b) Temperature-dependent normalized intensity ratio of *I*_Tb_ (^5^D_4_ → ^7^F_5_) to *I*_Eu_ (^5^D_0_ → ^7^F_2_) and the fitted curve for Tb_0.995_Eu_0.05_TPDB.


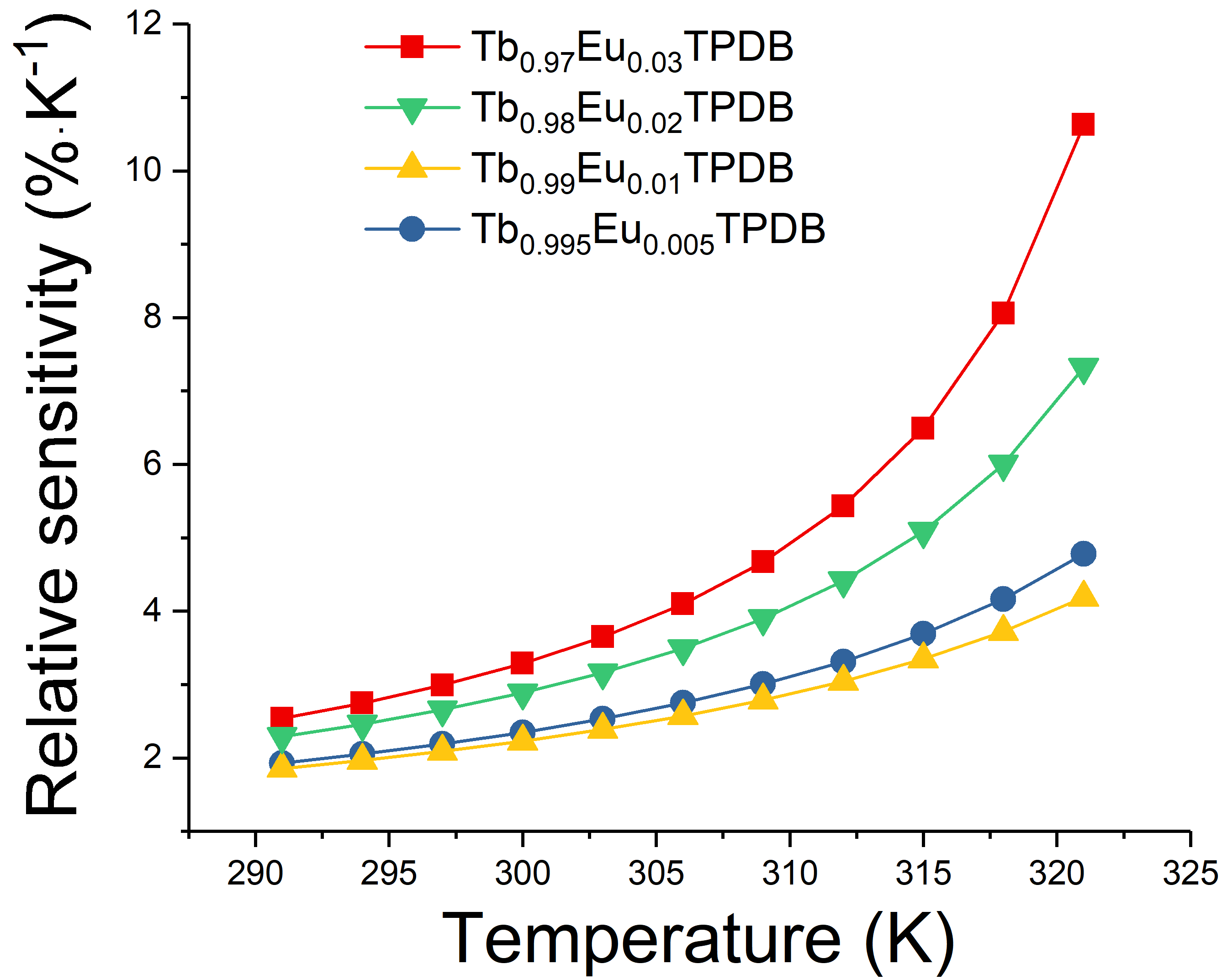


**Figure S14**. Comparison of the performance of ratio thermometer based on different ratios of Tb^3+^ and Eu^3+^.


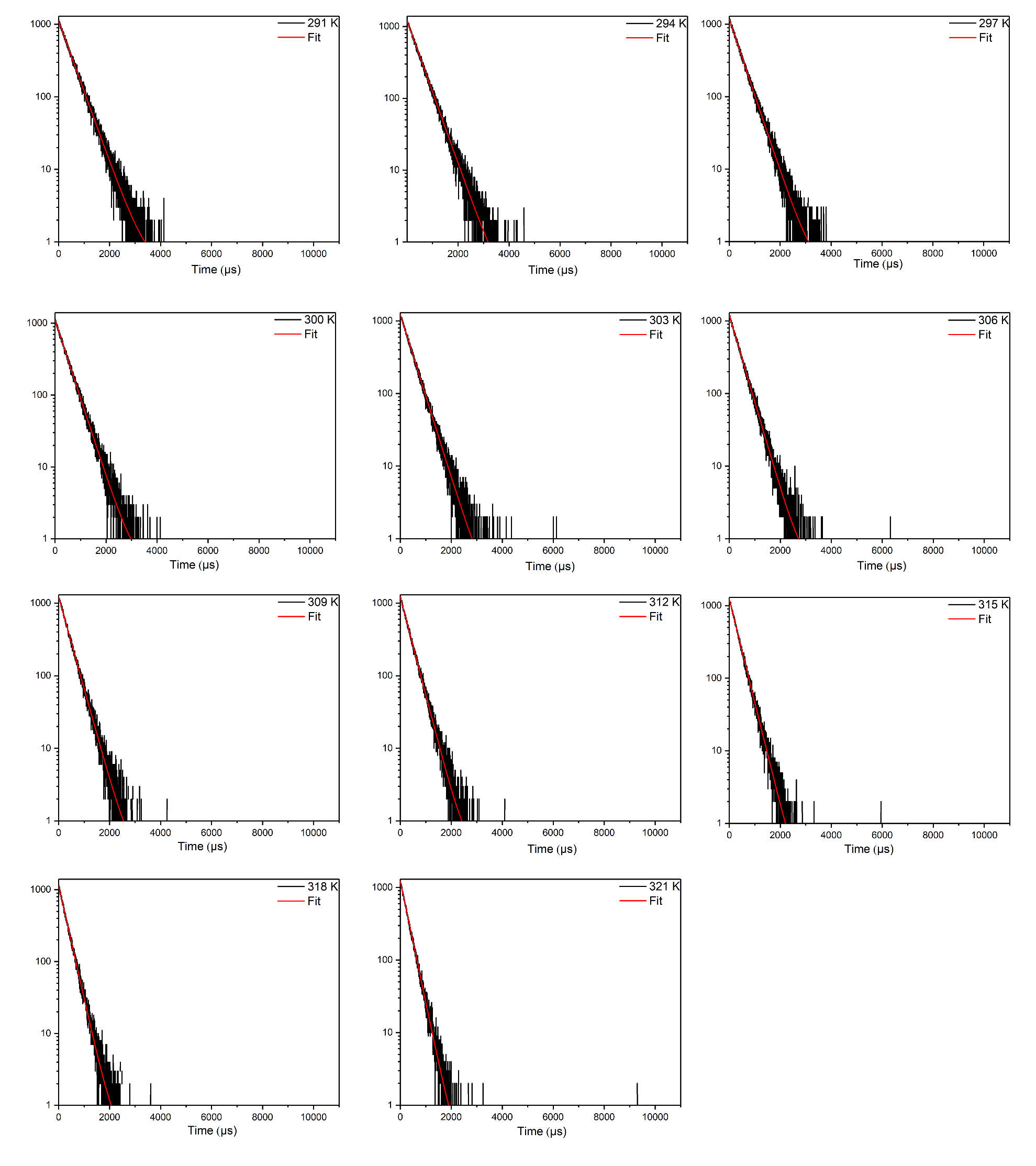


**Figure S15**. The luminescence decay curves of the ^5^D_4_ → ^7^F_5_ (542 nm, Tb^3+^) transition in Tb_0.98_Eu_0.02_TPDB from 291 K to 321 K.


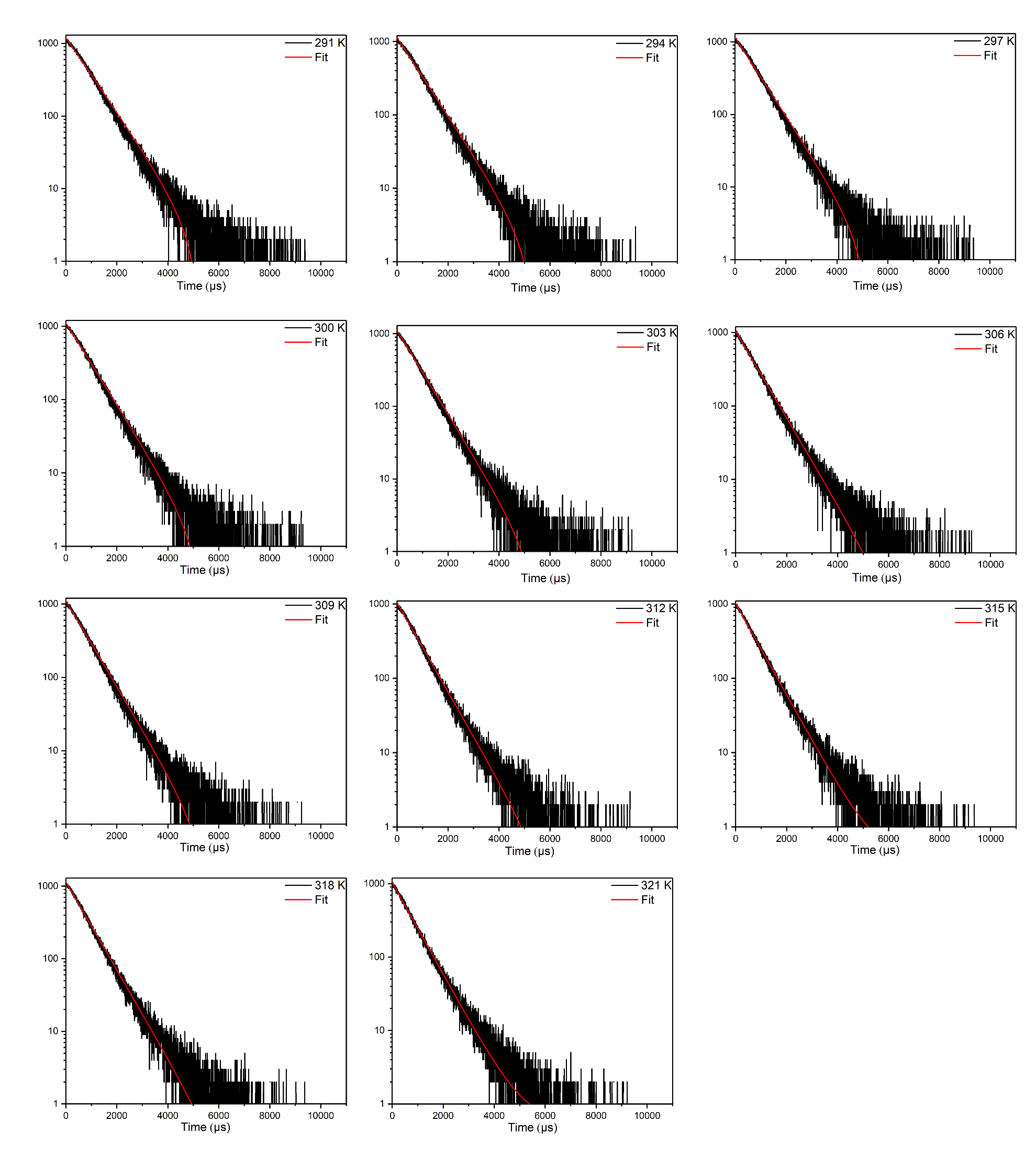


**Figure S16**. The luminescence decay curves of the ^5^D_0_ → ^7^F_2_ (615 nm, Eu^3+^) transition in Tb_0.98_Eu_0.02_TPDB from 291 K to 321 K.


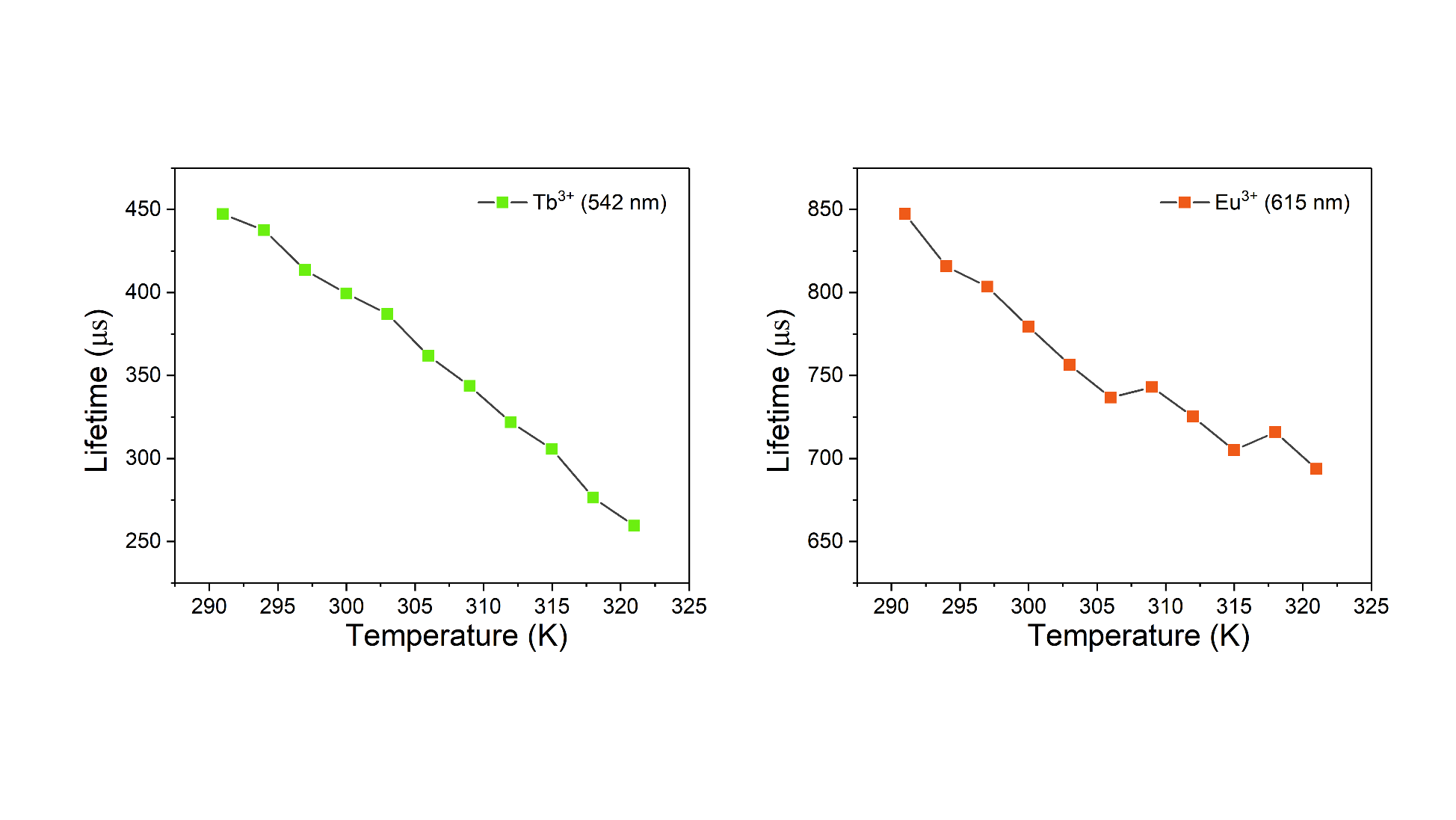


**Figure S17**. Temperature-dependence of the lifetimes of 542 nm for Tb^3+^ (542 nm) and Eu^3+^ (615 nm) emissions in Tb_0.98_Eu_0.02_TPDB.


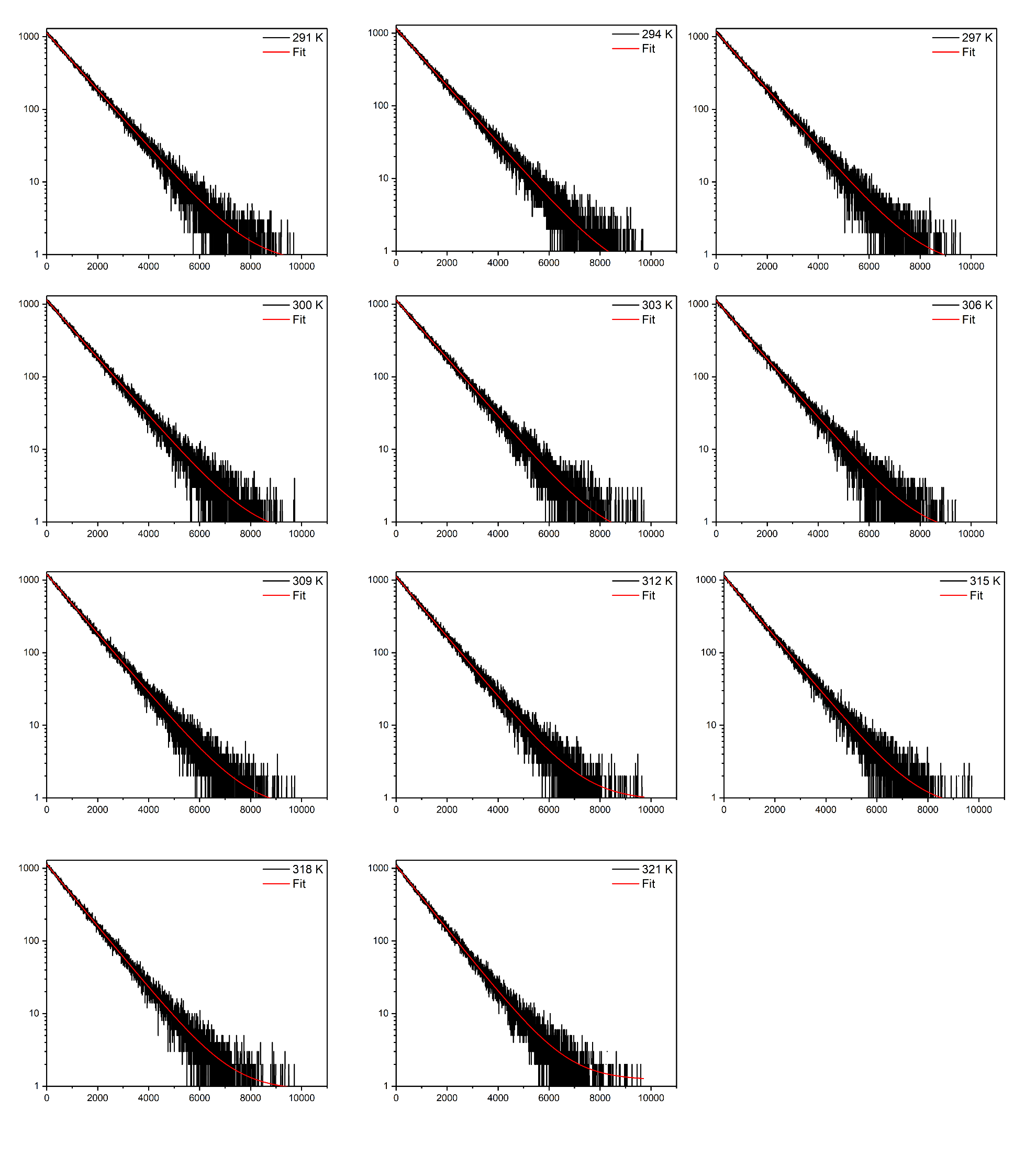


**Figure S18**. The luminescence decay curves of the ^5^D_4_ → ^7^F_5_ (542 nm, Tb^3+^) transition in TbTPDB from 291 K to 321 K.


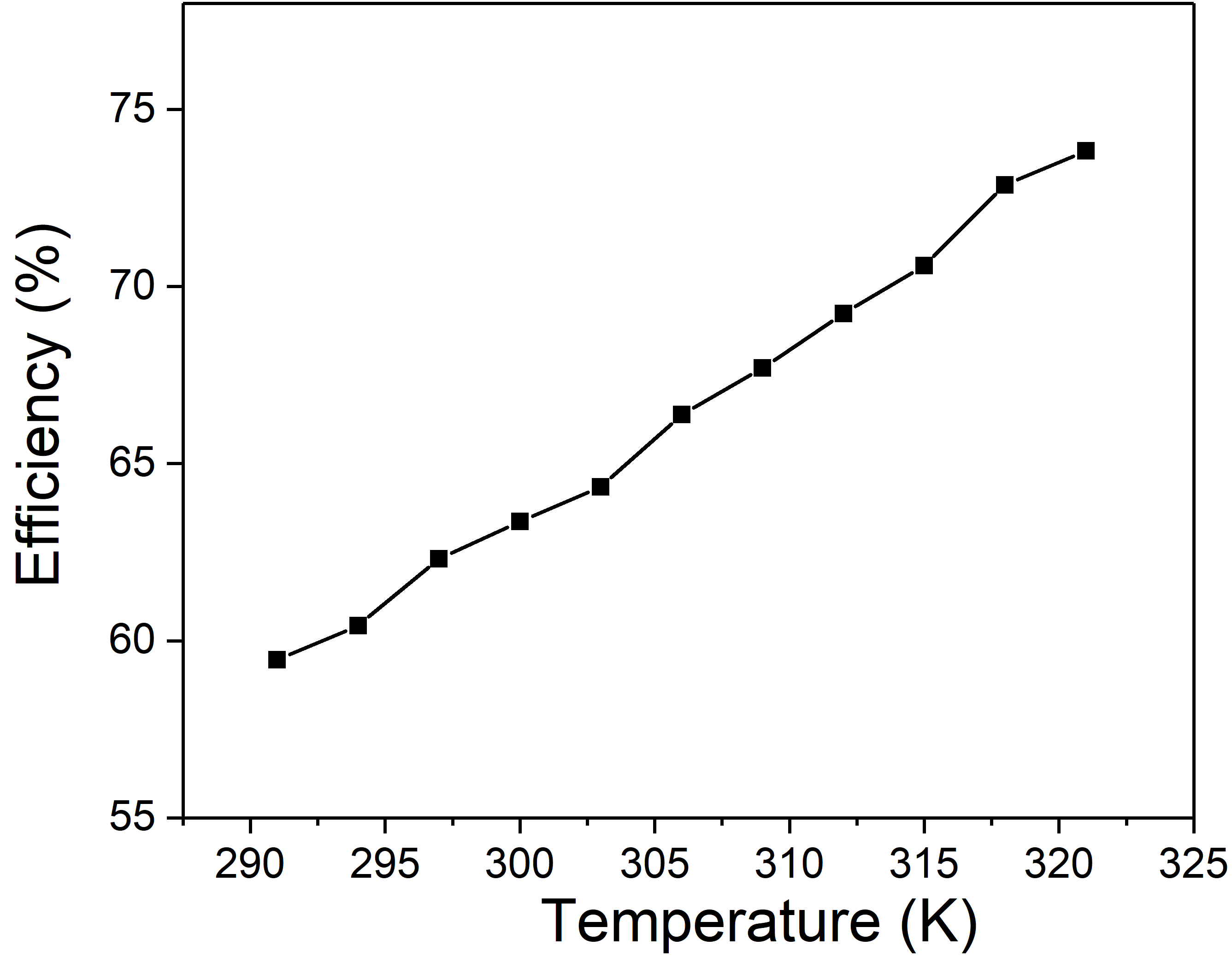


**Figure S19**. Temperature-dependent energy transfer efficiency from Tb^3+^ to Eu^3+^ ions in Tb_0.98_Eu_0.02_TPDB.


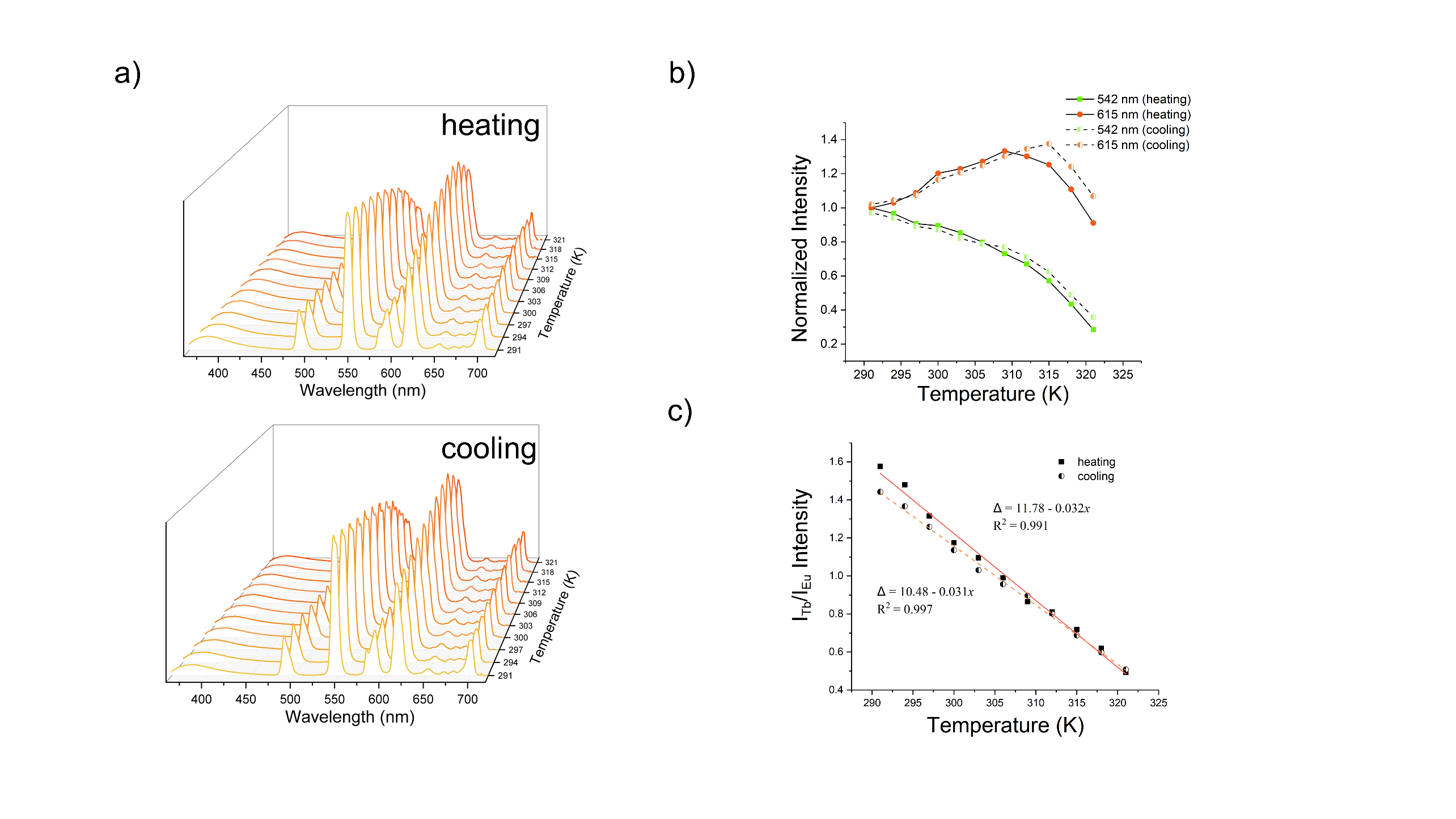


**Figure S20**. a) Emission spectra of Tb_0.98_Eu_0.02_TPDB recorded when heating and cooling (λ_ex_ = 338 nm). b) Temperature-dependence of the normalized intensity of 542 nm for Tb^3+^ (^5^D_4_ → ^7^F_5_) and 615 nm for Eu^3+^ (^5^D_0_ → ^7^F_2_) transitions for Tb_0.98_Eu_0.02_TPDB when heating and cooling. c) Temperature-dependent intensity ratio of 542 nm (Tb^3+^) to 615 nm (Eu^3+^) and the fitted curve for Tb_0.98_Eu_0.02_TPDB when heating and cooling.


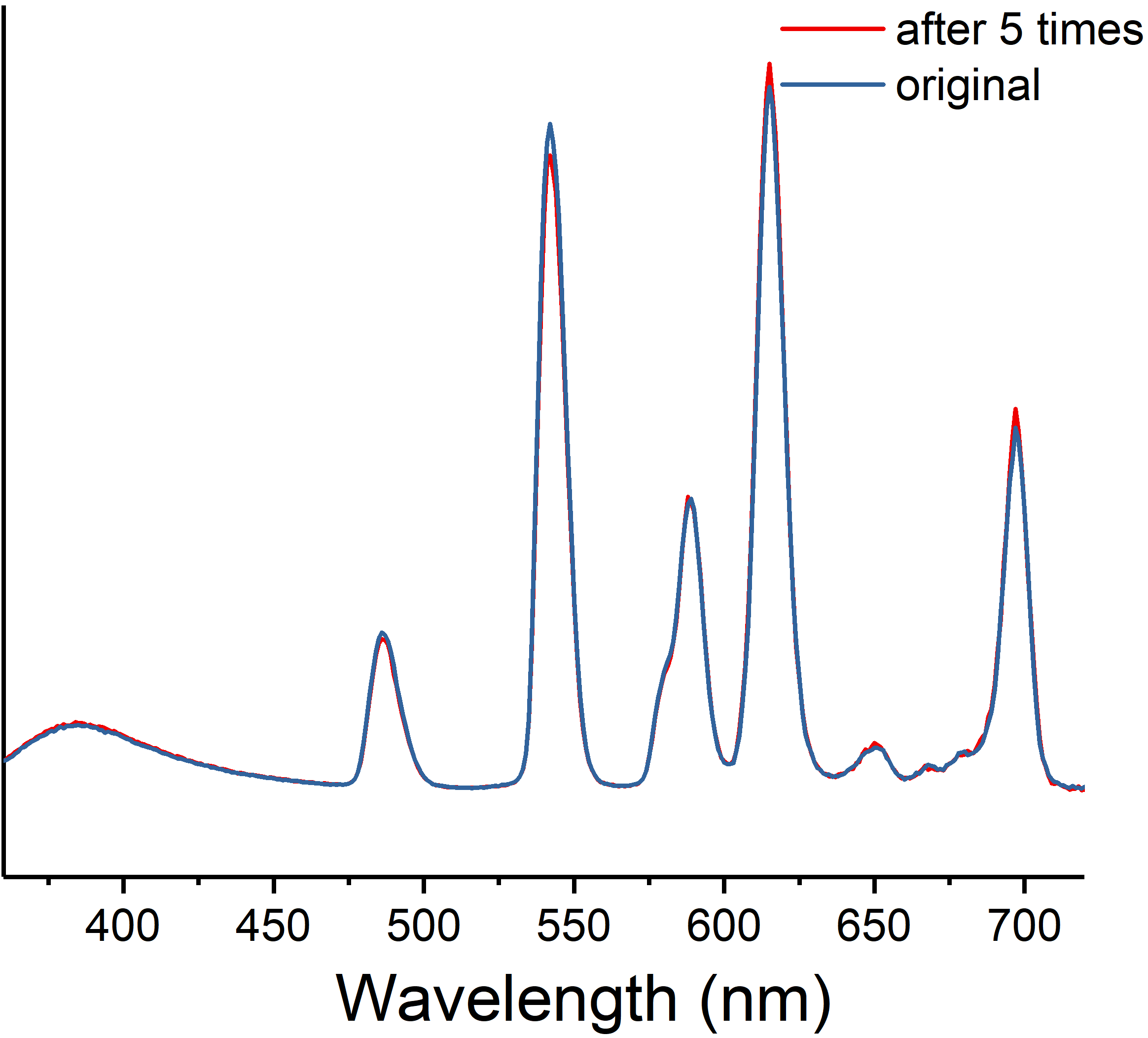


**Figure S21**. Emission spectra of the Tb_0.98_Eu_0.02_TPDB before and after 5 cycles of use ( λ_ex_ = 338 nm).


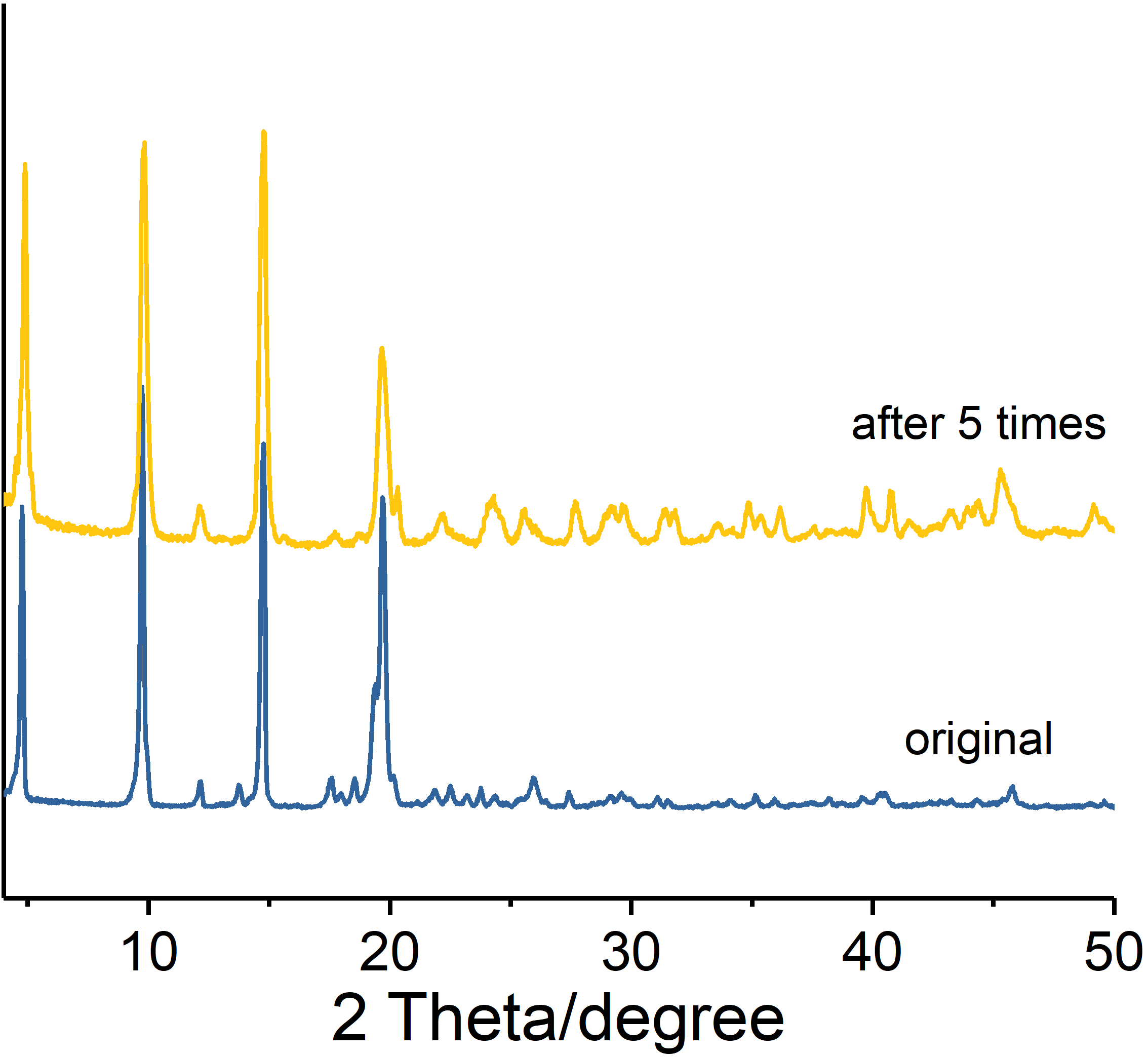


**Figure S22**. PXRD patterns of the Tb_0.98_Eu_0.02_TPDB before and after 5 cycles of use.


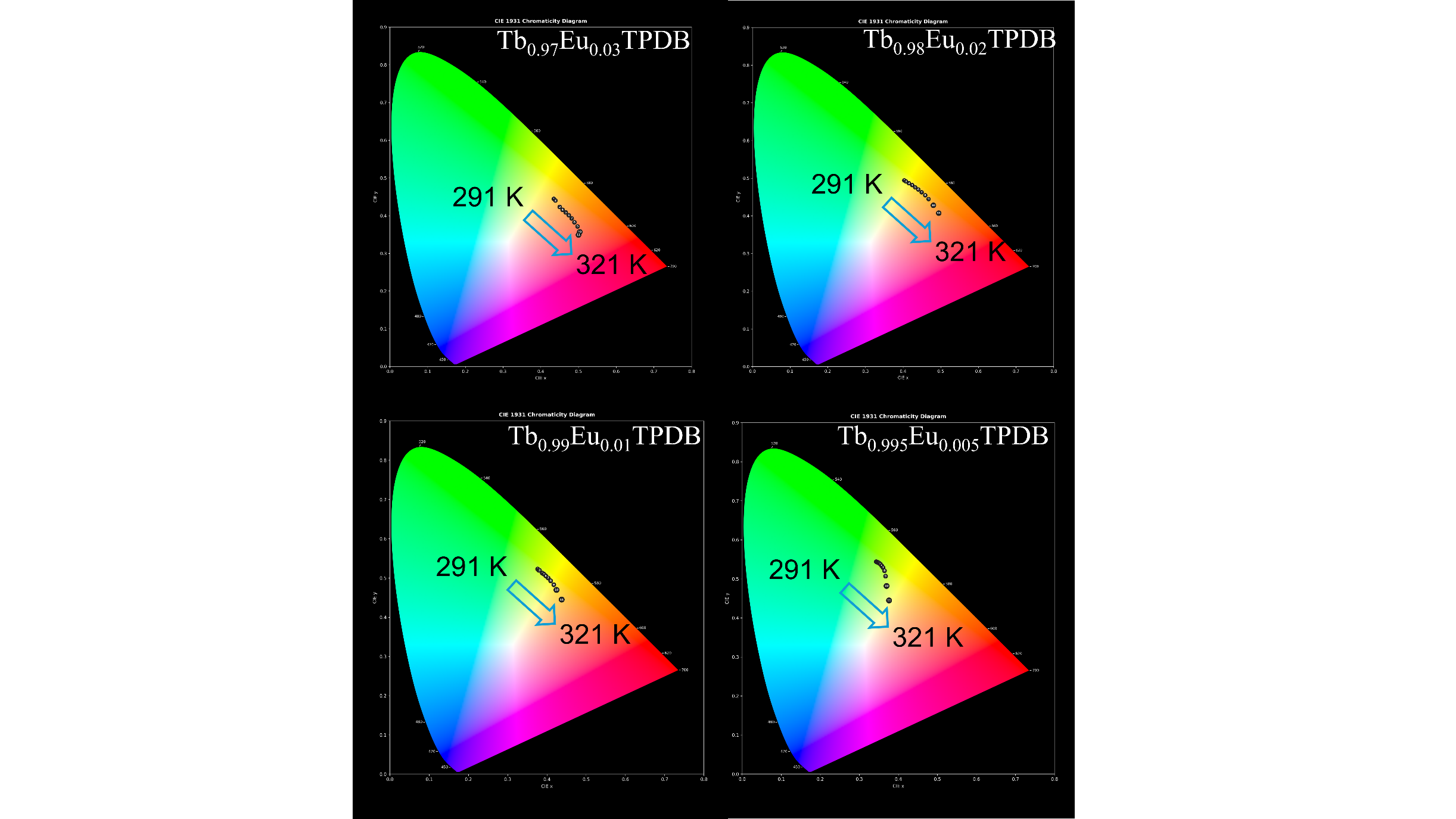


**Figure S23**. The chromaticity coordinate diagram showing the luminescence colors of Tb_1-_*_x_*Eu*_x_*TPDB at different temperatures.


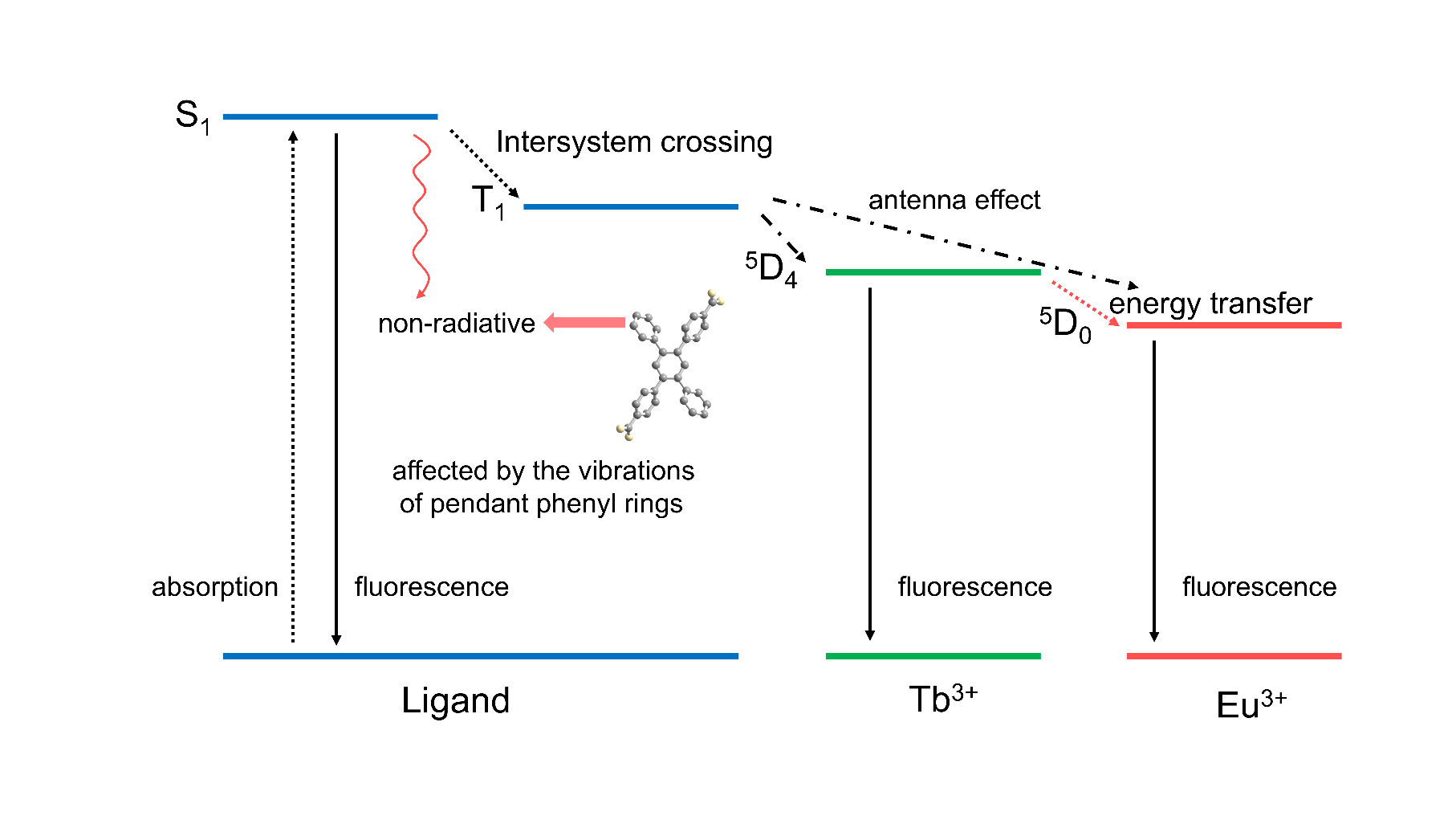


**Figure S24**. Multiple energy transfer processes in Tb_0.98_Eu_0.02_TPDB.


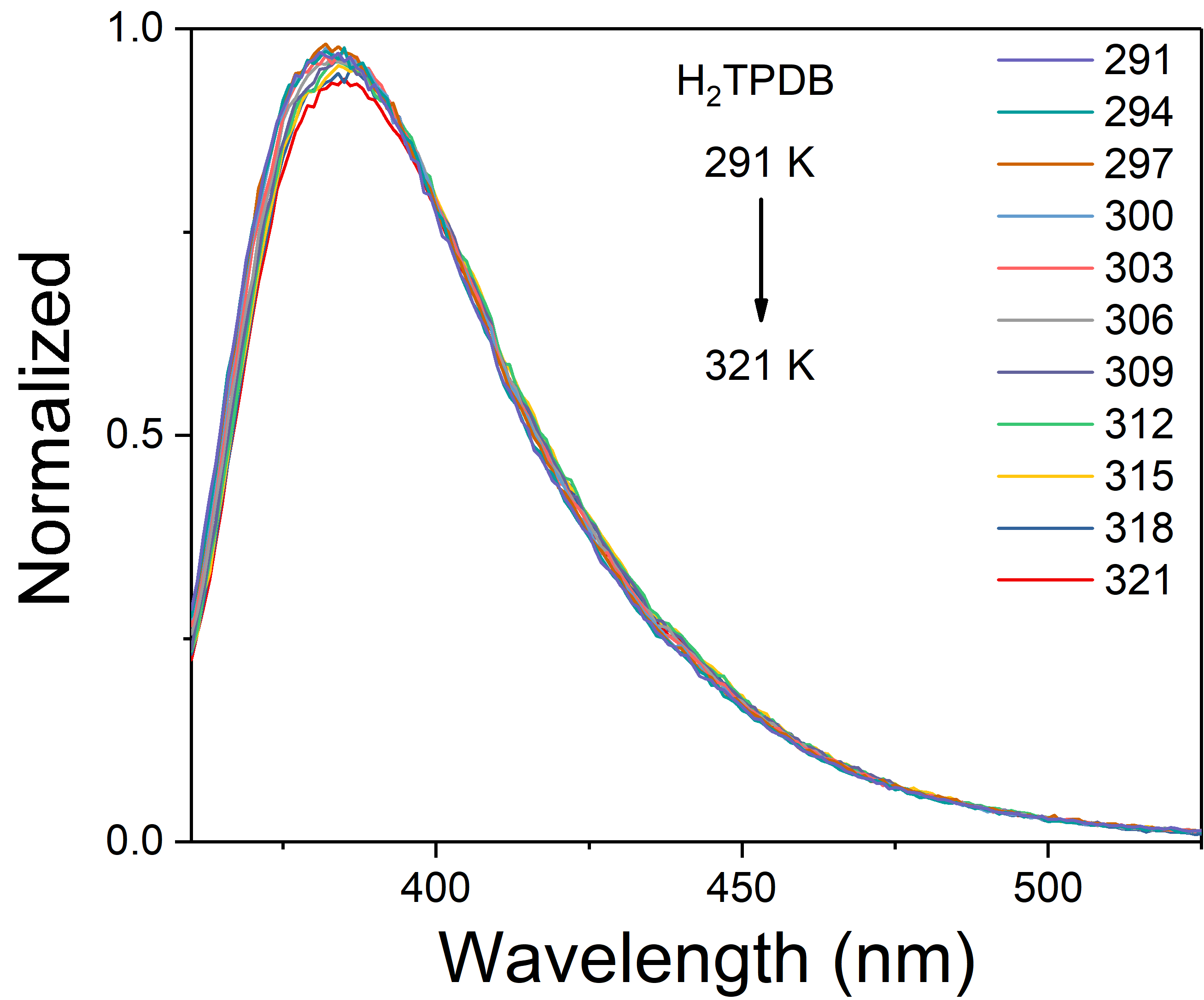


**Figure S25.** Emission spectra of H_2_TDPB recorded from 291 K to 321 K (λ_ex_ = 338 nm).


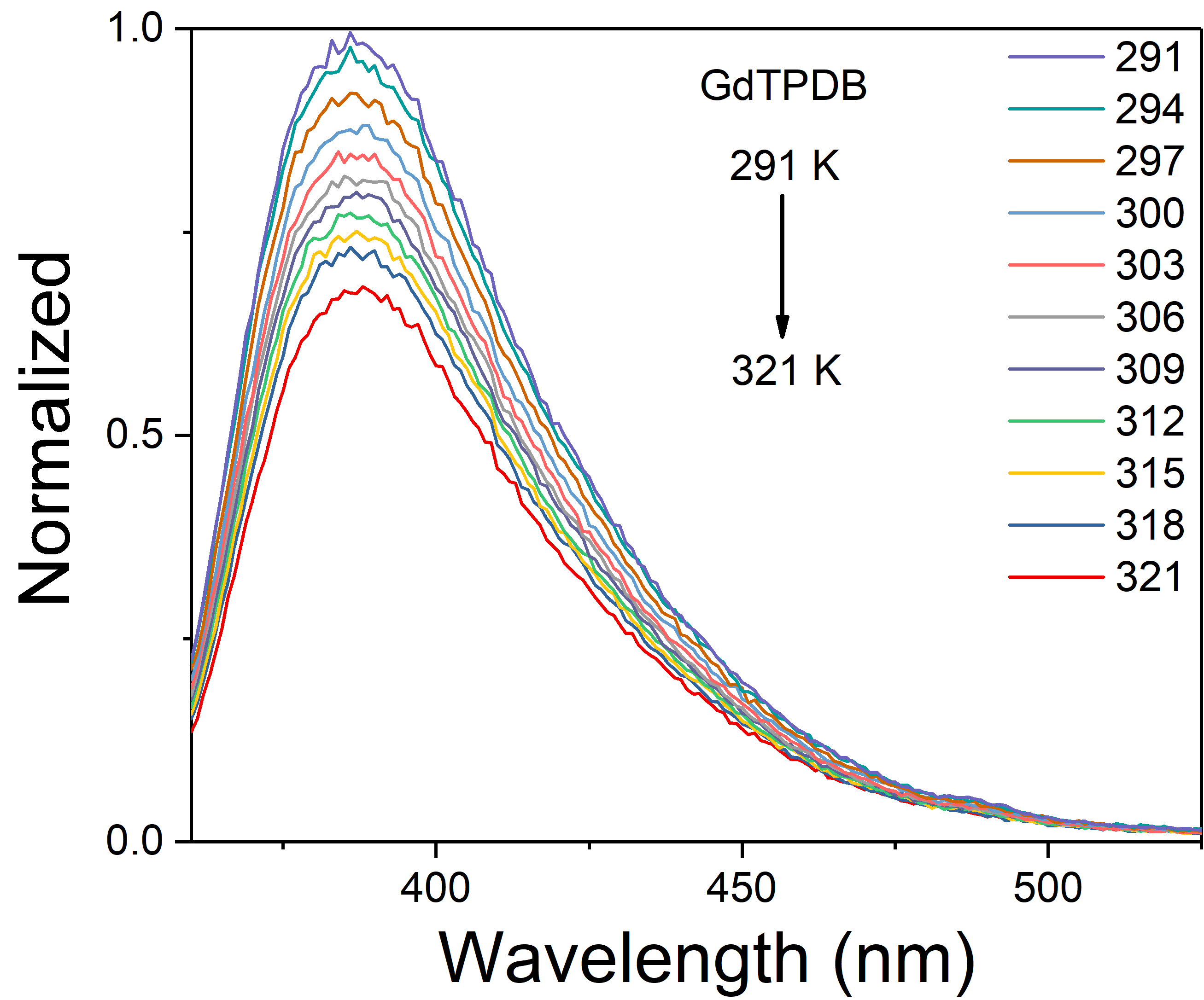


**Figure S26.** Emission spectra of GdTDPB recorded from 291 K to 321 K (λ_ex_ = 338 nm).


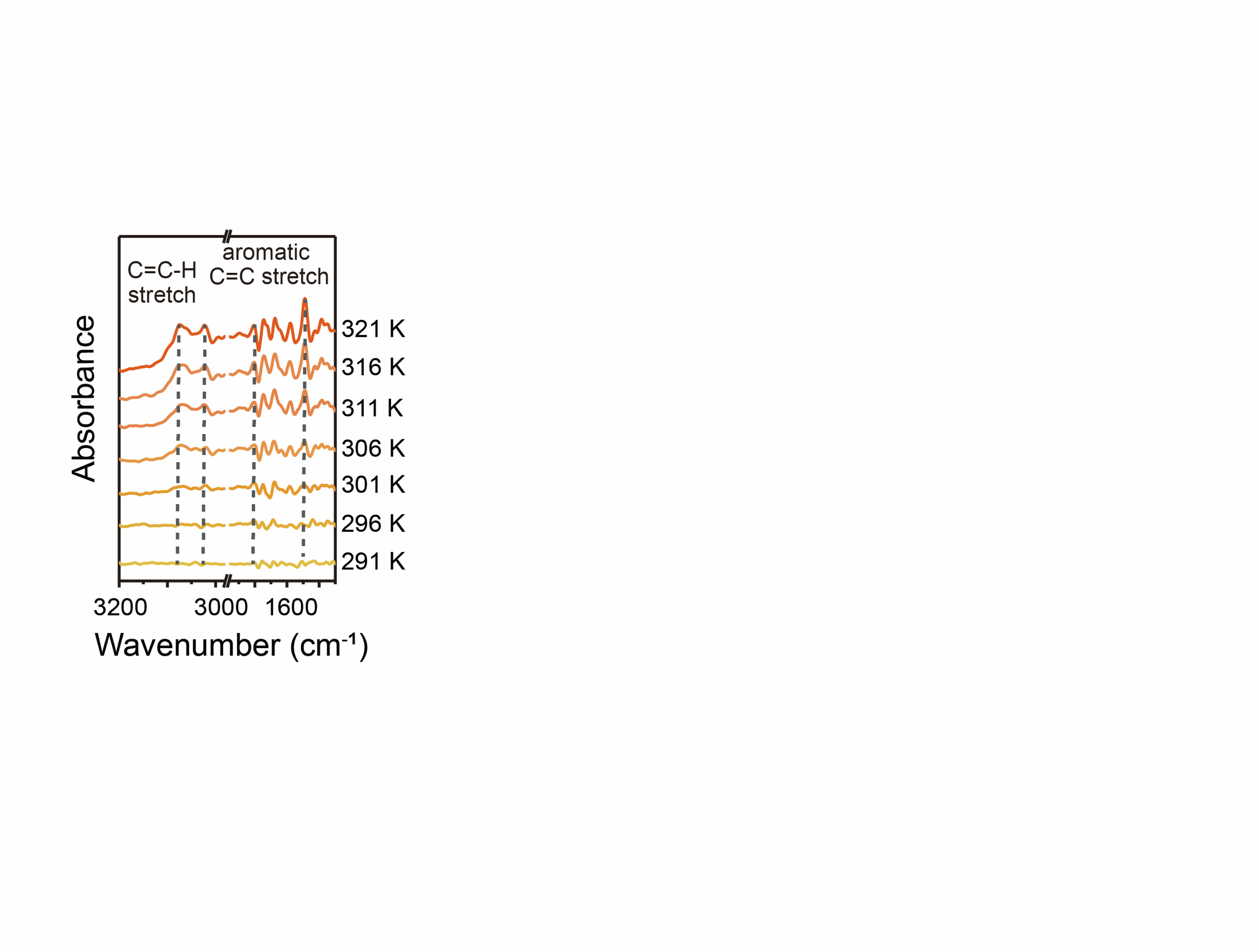


**Figure S27**. The *in situ* FT-IR spectra of Tb_0.98_Eu_0.02_TPDB from 291 K to 321 K.


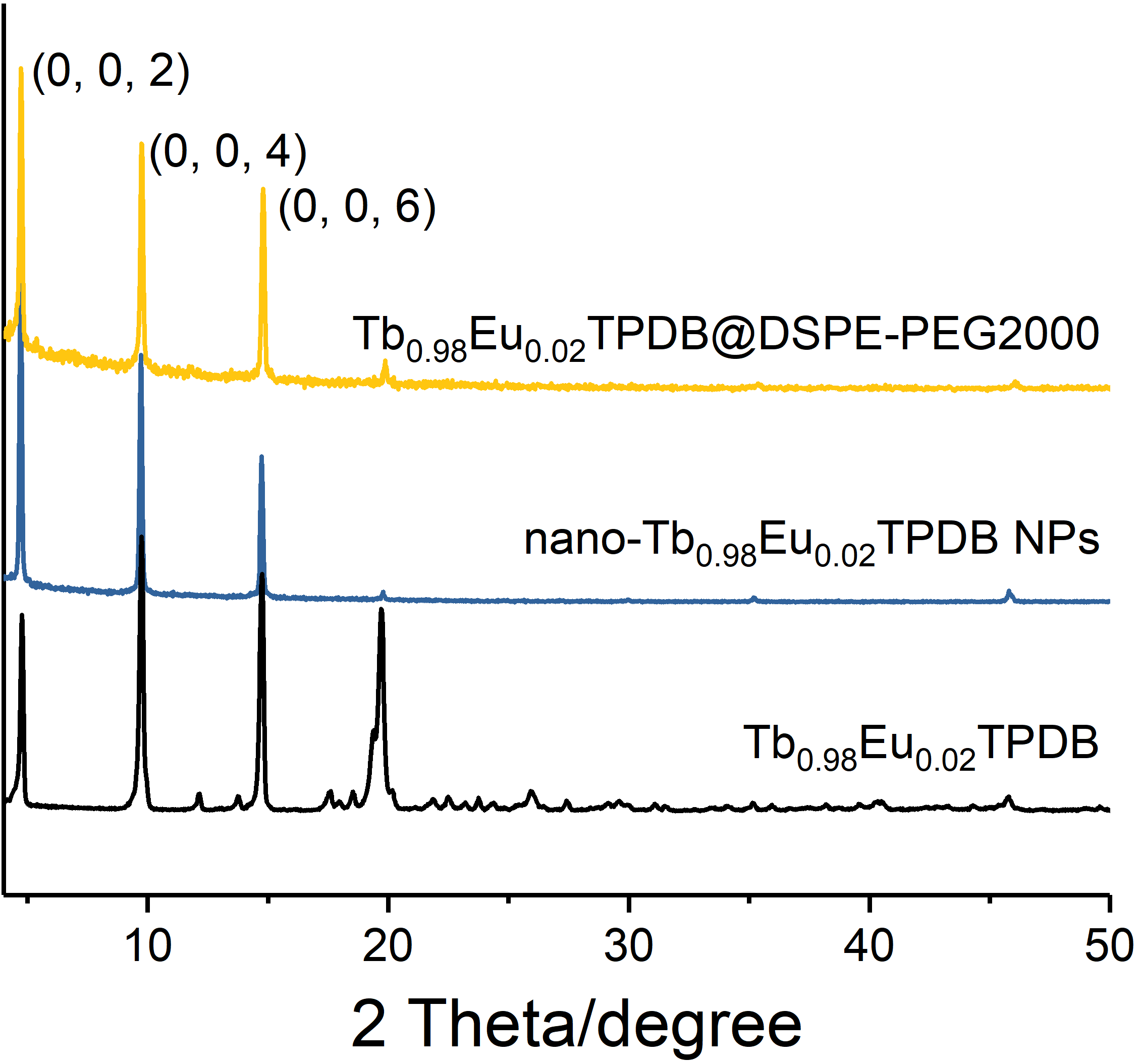


**Figure S28**. PXRD patterns of Tb_0.98_Eu_0.02_TPDB NPs and Tb_0.98_Eu_0.02_TPDB@DSPE-PEG2000.


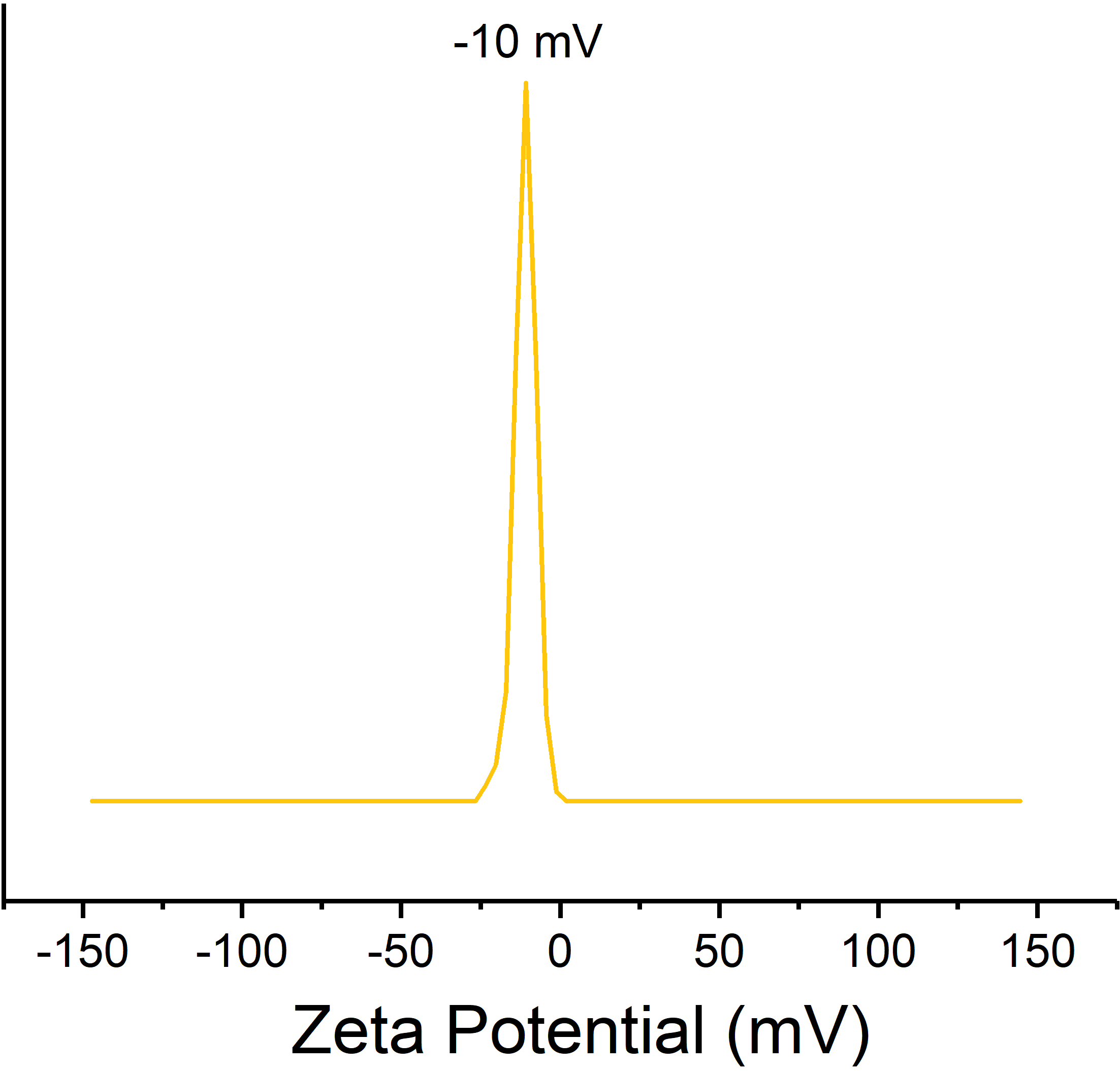


**Figure S29**. Zeta potential of Tb_0.98_Eu_0.02_TPDB@DSPE-PEG2000 in water.


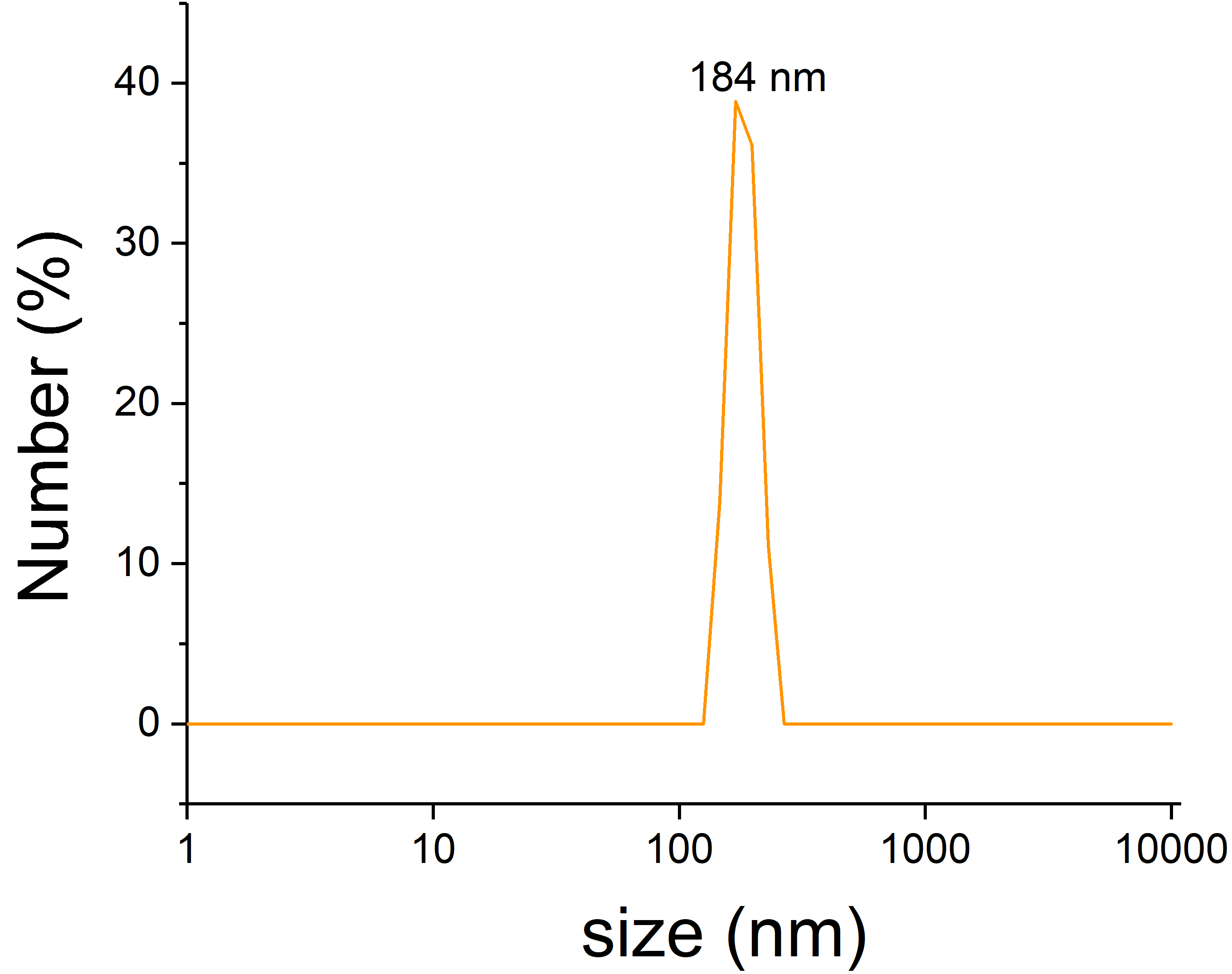


**Figure S30**. Size distribution of Tb_0.98_Eu_0.02_TPDB@DSPE-PEG2000 in water.


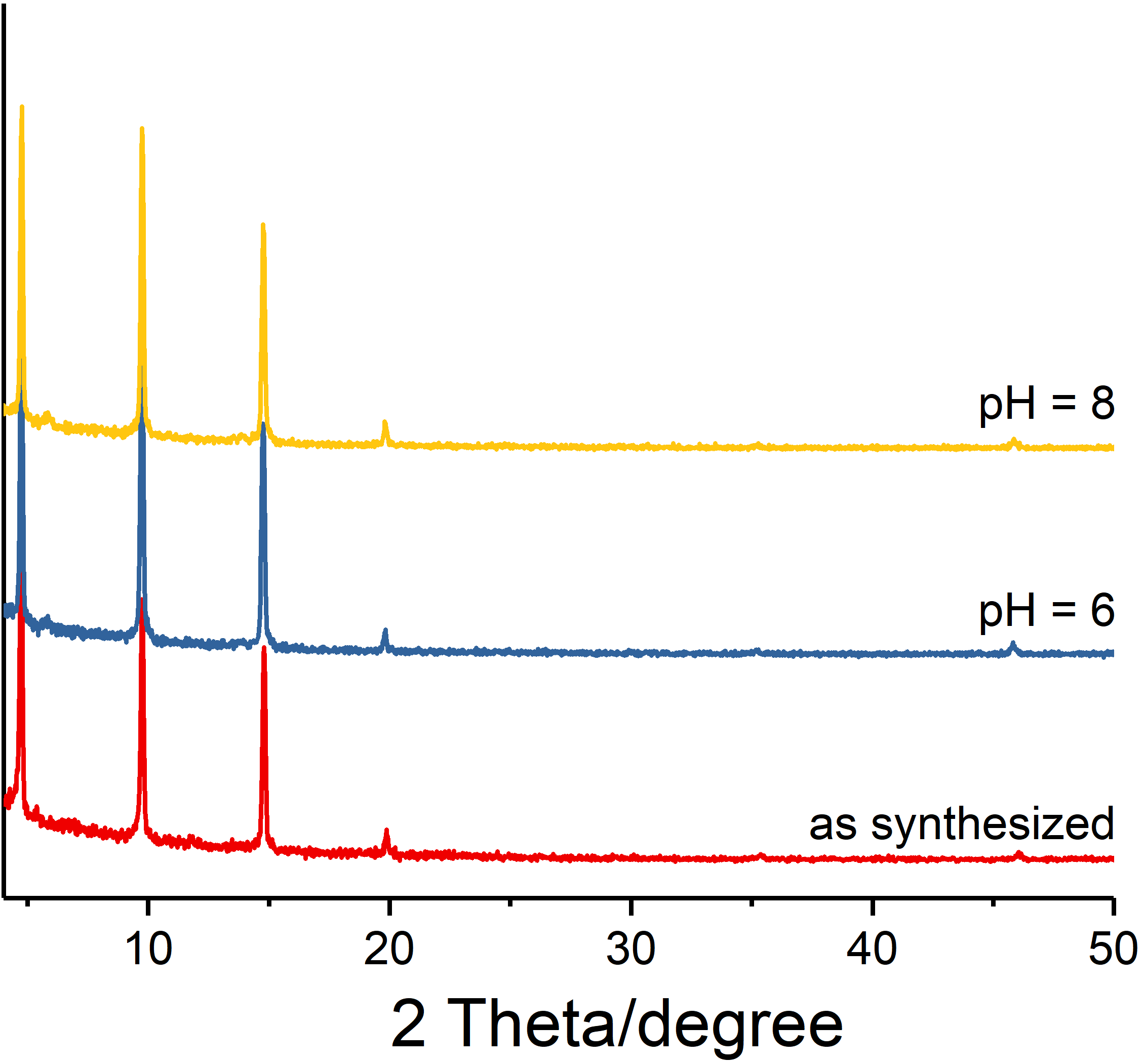


**Figure S31**. PXRD patterns of Tb_0.98_Eu_0.02_TPDB@DSPE-PEG2000 after being dispersed in different pH aqueous solutions (pH = 6 or 8) for 24 h.


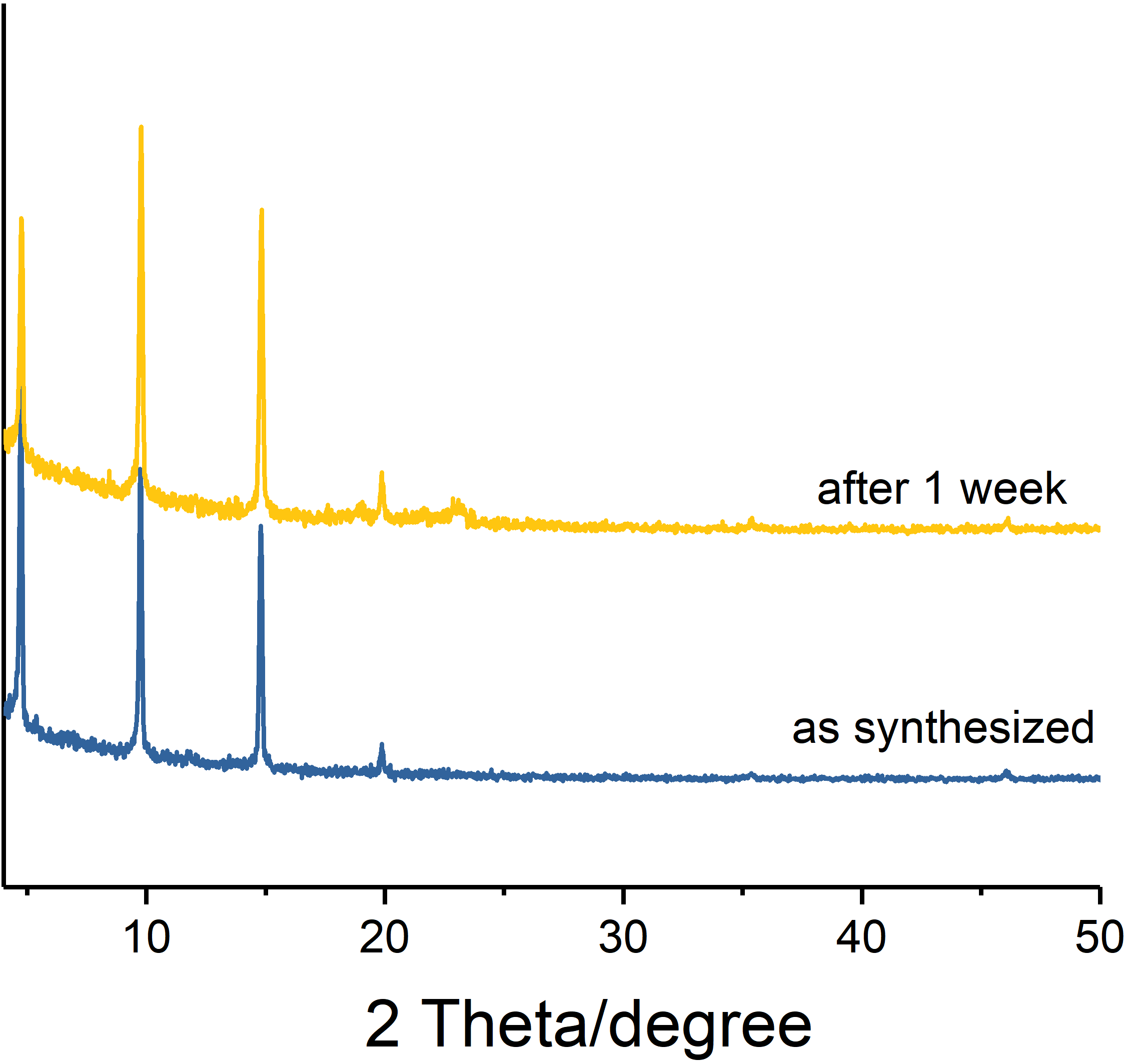


**Figure S32**. PXRD patterns of Tb_0.98_Eu_0.02_TPDB@DSPE-PEG2000 before and after being placed at room temperature for one week.


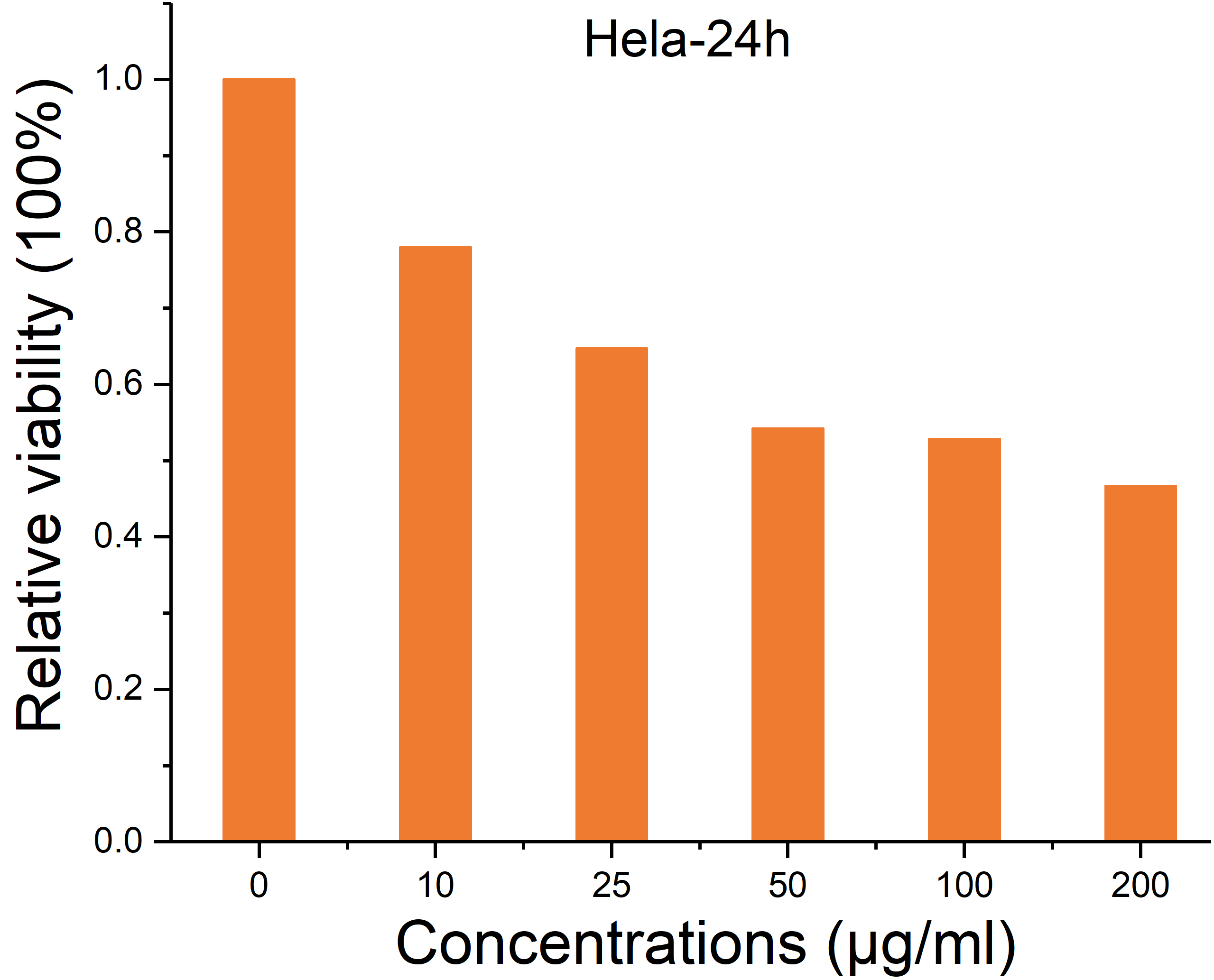


**Figure S33**. MTT assay of Tb_0.98_Eu_0.02_TPDB NPs.


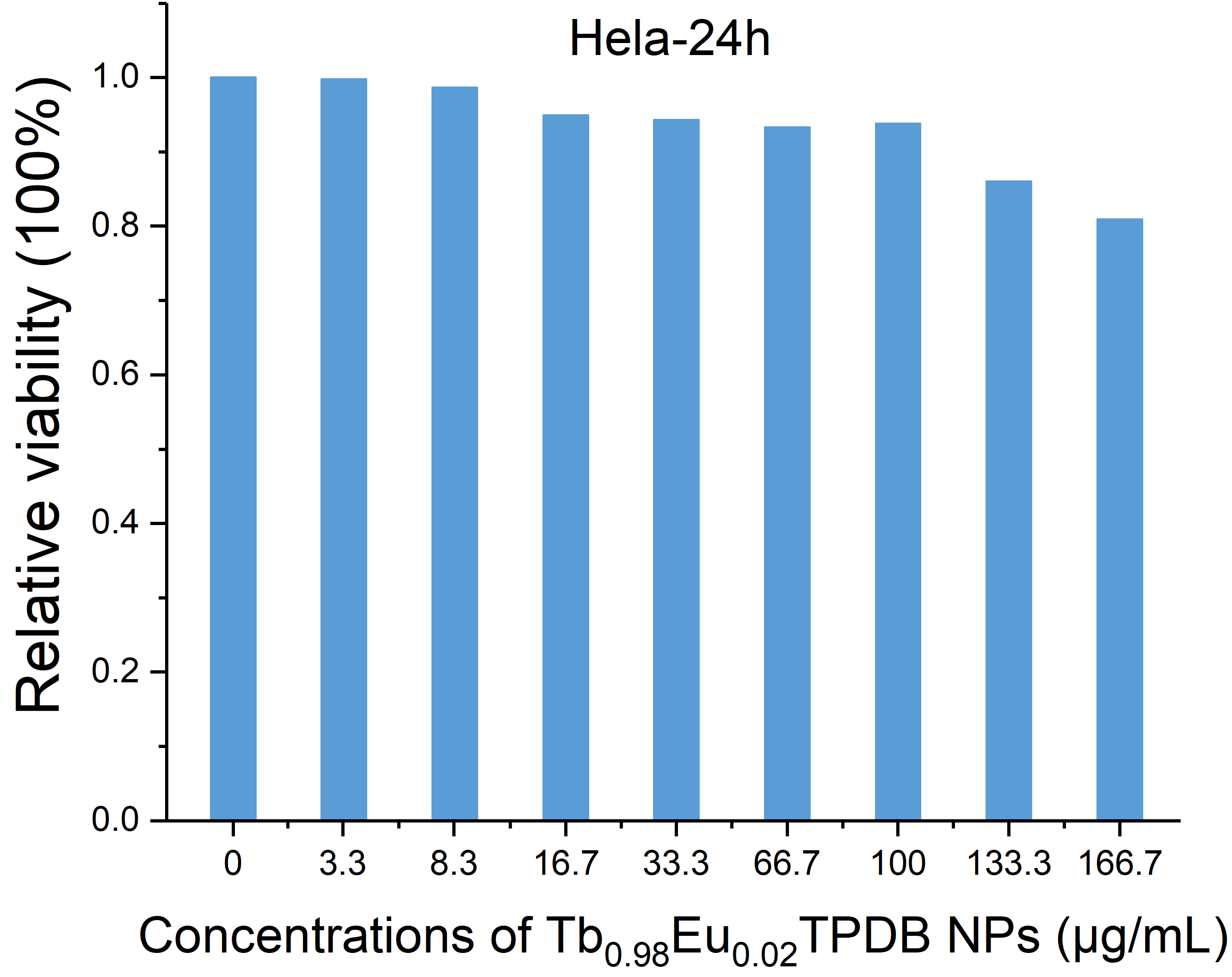


**Figure S34**. MTT assay of Tb_0.98_Eu_0.02_TPDB@DSPE-PEG2000 (the concentrations of Tb_0.98_Eu_0.02_TPDB NPs were used for comparison).


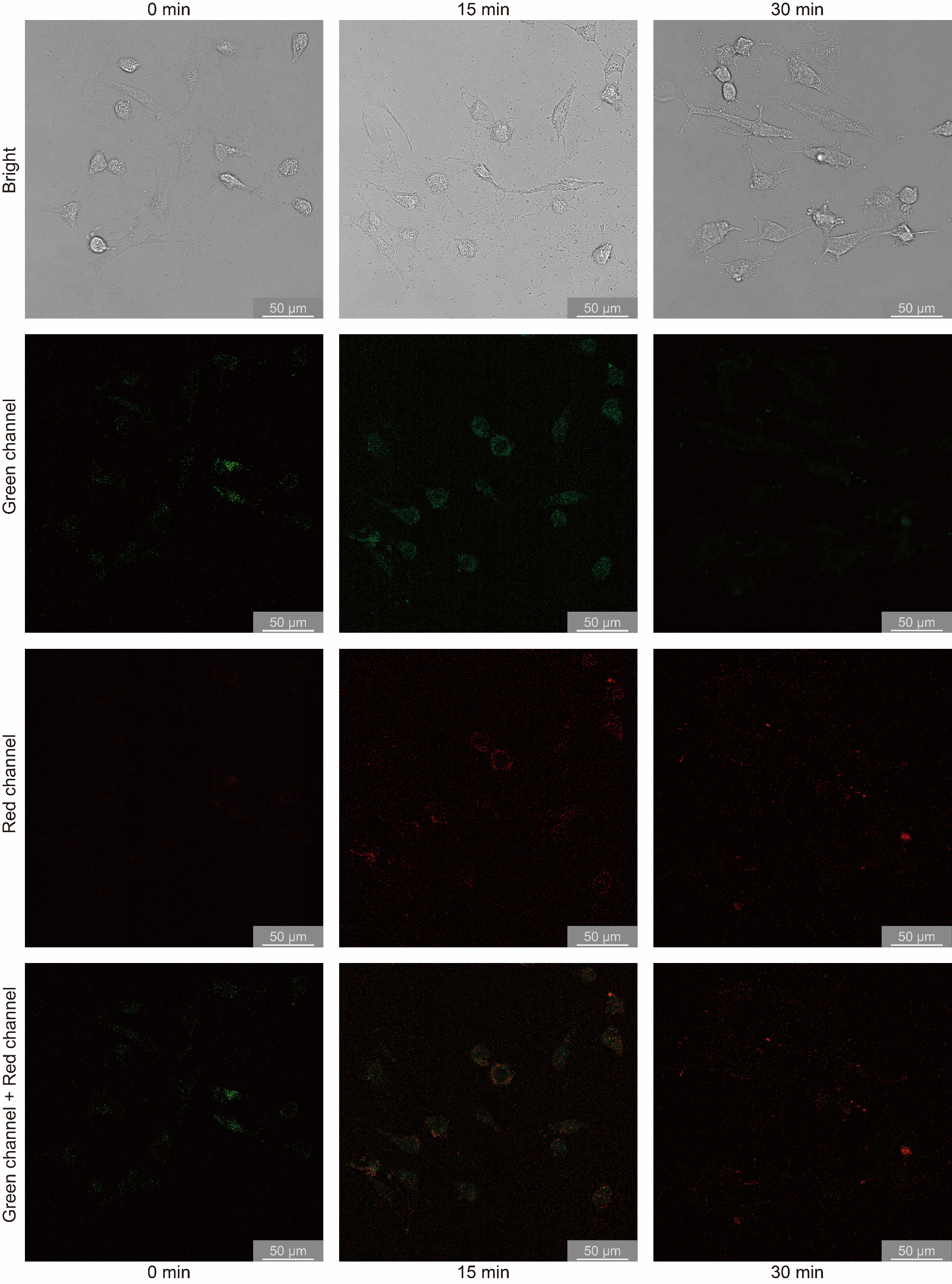


**Figure S35**. Confocal laser scanning microscopy images of Hela cells, treated with lipopolysaccharide for different times. Bright field images (the first row), green channel (the second row), red channel (the third row), green channel and red channel (the fourth row). Excitation wavelength: 405 nm.

**Table S1**. Crystal data and structure refinement parameters for TbTPDB

| Empirical formula | C_38_H_38_N_2_O_8_Tb |
| --- | --- |
| Formula weight | 809.62 |
| Crystal system | monoclinic |
| Space group | *I*2/a |
| a/Å | 10.4272(2) |
| b/Å | 8.85878(15) |
| c/Å | 35.7266(7) |
| α/° | 90 |
| β/° | 94.8834(19) |
| γ/° | 90 |
| Volume/Å^3^ | 3288.17(11) |
| Z | 4 |
| ρ_calc_g/cm^3^ | 1.635 |
| μ/mm^‑1^ | 11.060 |
| F(000) | 1636.0 |
| Reflections collected | 16233 |
| Independent reflections | 3346 [R_int_ = 0.0342, R_sigma_ = 0.0228] |
| Goodness-of-fit on F^2^ | 1.098 |
| Final R indexes [I>=2σ (I)] | R_1_ = 0.0391, wR_2_ = 0.1103 |
| Final R indexes [all data] | R_1_ = 0.0397, wR_2_ = 0.1109 |

**Table S2**. The actual ratios of Tb^3+^/Eu^3+^ in Tb_1-_*_x_*Eu*_x_*TPDB by ICP analysis.

| Samples | Tb^3+^ | Eu^3+^ |
| --- | --- | --- |
| Tb_0.97_Eu_0.03_TPDB | 0.97194 | 0.02806 |
| Tb_0.98_Eu_0.02_TPDB | 0.98025 | 0.01975 |
| Tb_0.99_Eu_0.01_TPDB | 0.98961 | 0.01039 |
| Tb_0.995_Eu_0.005_TPDB | 0.99369 | 0.00631 |
| Tb_0.98_Eu_0.02_TPDB NPs | 0.98009 | 0.01971 |

**Table S3**. The QYs of LnTPDB and Tb_1-_*_x_*Eu*_x_*TPDB.

| Samples | *Φ*F (%) |
| --- | --- |
| TbTPDB | 54.30 |
| EuTPDB | 27.43 |
| Tb_0.97_Eu_0.03_TPDB | 41.52 |
| Tb_0.98_Eu_0.02_TPDB | 46.41 |
| Tb_0.99_Eu_0.01_TPDB | 47.45 |
| Tb_0.995_Eu_0.005_TPDB | 55.39 |

**Table S4**. Comparison of the performance of ratio thermometer based on MOFs in terms of temperature range^[2–12]^.

| MOF | Range (K) | S_m_ (%K^-1^) | T_m_ (K) | Ref. |
| --- | --- | --- | --- | --- |
| Tb_0.9_Eu_0.1_PIA | 100-300 | 3.27 | 300 | 1 |
| Tb_0.99_Eu_0.01_(BDC)_1.5_·(H_2_O)_2_ | 290-320 | 0.31 | 318 | 2 |
| Eu_0.0069_Tb_0.9931_-DMBDC | 50-200 | 1.15 | 200 | 3 |
| Tb_0.957_Eu_0.043_cpda | 40-300 | 16 | 300 | 4 |
| [(Tb_0.9_^[1–8]^_14_Eu_0.086_)_2_(pda)_3_(H2O)]·H_2_O | 10-325 | 5.96 | 25 | 5 |
| Tb_0.80_Eu_0.20_(bpda) | 303-328 | 1.39 | 328 | 6 |
| Eu_0.0025_Tb_0.9975_-BABDC | 90-240 | 3.74 | 240 | 7 |
| ZJU-88⊃perylene | 293-353 | 1.27 | 353 | 8 |
| Nd_0.676_Yb_0.324_BTC | 288-323 | 1.187 | 323 | 9 |
| Nd_0.866_Yb_0.134_BTB | 303-333 | 4.755 | 333 | 10 |
| Eu_0.058_Tb_0.942_BPT | 293-353 | 7.22 | 353 | 11 |
| Tb_0.98_Eu_0.02_TPDB | 291-321 | 7.32 | 321 | This work |

**Table S5**. Temperature-dependent lifetimes of Tb_0.98_Eu_0.02_TPDB.

| Temperature (K) | *τ* (542 nm) (μs) | *τ* (615 nm) (μs) |
| --- | --- | --- |
| 291 | 447.13 | 847.24 |
| 294 | 437.59 | 815.79 |
| 297 | 413.46 | 803.38 |
| 300 | 399.35 | 779.41 |
| 303 | 387.04 | 756.34 |
| 306 | 361.82 | 736.72 |
| 309 | 343.55 | 742.97 |
| 312 | 321.74 | 725.17 |
| 315 | 305.73 | 704.91 |
| 318 | 276.39 | 715.92 |
| 321 | 259.44 | 693.62 |

**Table S6**. Temperature-dependent lifetimes of TbTPDB.

| Temperature (K) | *τ* (542 nm) (μs) |
| --- | --- |
| 291 | 1103.14 |
| 294 | 1105.81 |
| 297 | 1097.14 |
| 300 | 1090.02 |
| 303 | 1085.50 |
| 306 | 1076.30 |
| 309 | 1063.78 |
| 312 | 1045.98 |
| 315 | 1039.33 |
| 318 | 1018.91 |
| 321 | 991.23 |

**References**

[1] Z. Li, F. Jiang, M. Yu, S. Li, L. Chen, M. Hong, *Nat. Commun.* **2022**, *13*, 2142.

[2] X. Rao, T. Song, J. Gao, Y. Cui, Y. Yang, C. Wu, B. Chen, G. Qian, *J. Am. Chem. Soc.* **2013**, *135*, 15559.

[3] A. Cadiau, C. D. S. Brites, P. M. F. J. Costa, R. A. S. Ferreira, J. Rocha, L. D. Carlos, *ACS Nano* **2013**, *7*, 7213.

[4] Y. Cui, H. Xu, Y. Yue, Z. Guo, J. Yu, Z. Chen, J. Gao, Y. Yang, G. Qian, B. Chen, *J. Am. Chem. Soc.* **2012**, *134*, 3979.

[5] Y. Cui, W. Zou, R. Song, J. Yu, W. Zhang, Y. Yang, G. Qian, *Chem. Commun.* **2014**, *50*, 719.

[6] Z. Wang, D. Ananias, A. Carné‐Sánchez, C. D. S. Brites, I. Imaz, D. Maspoch, J. Rocha, L. D. Carlos, *Adv. Funct. Mater.* **2015**, *25*, 2824.

[7] D. Zhao, X. Rao, J. Yu, Y. Cui, Y. Yang, G. Qian, *Inorg. Chem.* **2015**, *54*, 11193.

[8] T. Feng, Y. Ye, X. Liu, H. Cui, Z. Li, Y. Zhang, B. Liang, H. Li, B. Chen, *Angew. Chem. Int. Ed.* **2020**, *59*, 21752.

[9] Y. Cui, R. Song, J. Yu, M. Liu, Z. Wang, C. Wu, Y. Yang, Z. Wang, B. Chen, G. Qian, *Adv. Mater.* **2015**, *27*, 1420.

[10] D. Yue, J. Zhang, D. Zhao, X. Lian, Y. Cui, Y. Yang, G. Qian, *J. Solid State Chem.* **2016**, *241*, 99.

[11] D. Zhao, J. Zhang, D. Yue, X. Lian, Y. Cui, Y. Yang, G. Qian, *Chem. Commun.* **2016**, *52*, 8259.

[12] L. Zhang, Y. Xie, T. Xia, Y. Cui, Y. Yang, G. Qian, *J. Rare Earths.* **2018**, *36*, 561.
